# Corrosion Inhibition Mechanism of Steel Reinforcements in Mortar Using Soluble Phosphates: A Critical Review

**DOI:** 10.3390/ma14206168

**Published:** 2021-10-18

**Authors:** David M. Bastidas, Ulises Martin, Jose M. Bastidas, Jacob Ress

**Affiliations:** 1 National Center for Education and Research on Corrosion and Materials Performance—NCERCAMP-UA, Department of Chemical, Biomolecular and Corrosion Engineering, The University of Akron, 302 E Buchtel Ave., Akron, OH 44325-3906, USA; um11@uakron.edu (U.M.); jtr45@uakron.edu (J.R.); 2 National Center for Metallurgical Research—CENIM, Consejo Superior de Investigaciones Científicas—CSIC, Ave. Gregorio del Amo 8, 28040 Madrid, Spain; bastidas@cenim.csic.es

**Keywords:** steel reinforcements, concrete, migrating corrosion inhibitors, phosphate penetration, thermodynamics, reactivity

## Abstract

The corrosion inhibition mechanism of soluble phosphates on steel reinforcement embedded in mortar fabricated with ordinary Portland cement (OPC) are reviewed. This review focuses soluble phosphate compounds, sodium monofluorophosphate (Na_2_PO_3_F) (MFP), disodium hydrogen phosphate (Na_2_HPO_4_) (DHP) and trisodium phosphate (Na_3_PO_4_) (TSP), embedded in mortar. Phosphate corrosion inhibitors have been deployed in two different ways, as migrating corrosion inhibitors (MCI), or as admixed corrosion inhibitors (ACI). The chemical stability of phosphate corrosion inhibitors depends on the pH of the solution, H_2_PO_4_^−^ ions being stable in the pH range of 3–6, the HPO_4_^2−^ in the pH range of 8–12, while the PO_4_^3−^ ions are stable above pH 12. The formation of iron phosphate compounds is a thermodynamically favored spontaneous reaction. Phosphate ions promote ferrous phosphate precipitation due to the higher solubility of ferric phosphate, thus producing a protective barrier layer that hinders corrosion. Therefore, the MFP as well as the DHP and TSP compounds are considered anodic corrosion inhibitors. Both types of application (MCI and ACI) of phosphate corrosion inhibitors found MFP to present the higher inhibition efficiency in the following order MFP > DHP > TSP.

## 1. Introduction

Due to its comparatively low cost and versatility, reinforced concrete is commonly used in the construction industry [1,2,3,4,5,6]. Despite its excellent compressive force, concrete alone is unable to withstand the necessary tensile load and reinforcements are necessary. The combined properties of concrete and steel reinforcements provide high compression strength as well as increased mechanical properties, thus making it an ideal composite material for a multitude of applications and structures [2]. The corrosion of these steel reinforcements is considered to be the greatest threat to the integrity of these structures and their service life [3,4]. Various solutions have been implemented to deter corrosion, such as corrosion inhibitors and many others [6,7,8,9,10].

**Figure 1:**
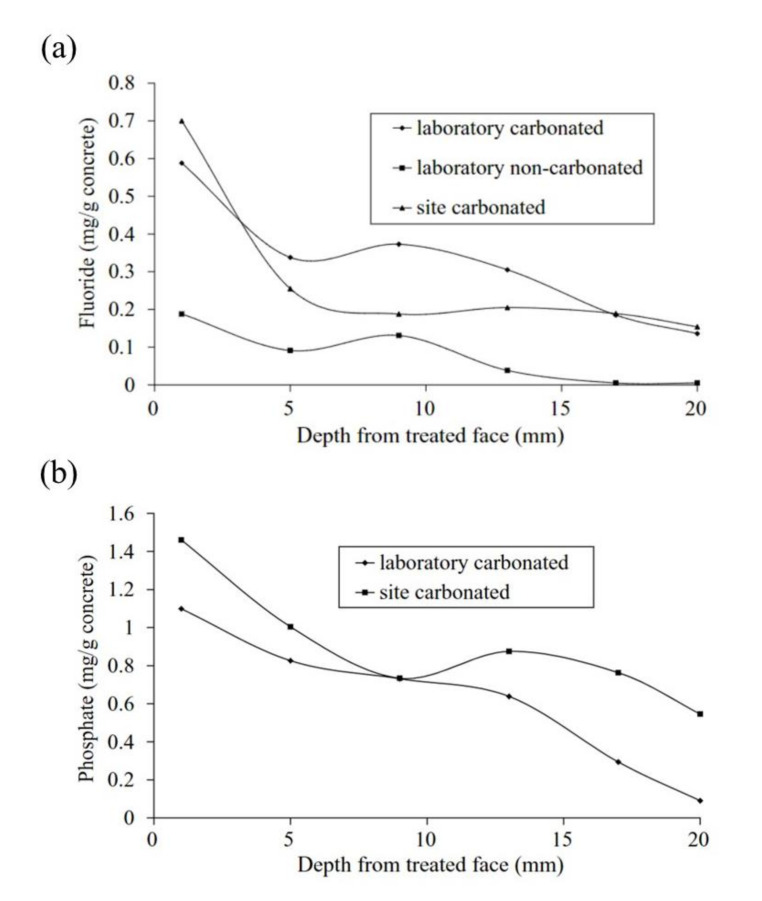
Penetration depth of inhibitors acting as migrating corrosion inhibitors (MCI): (**a**) water-soluble fluoride and (**b**) water-soluble phosphate [15]. Reproduced with permission from Ngala, V. et al., Corros. Sci.; published by Elsevier, 2003.

Corrosion inhibitors for steel in concrete can be used by addition to the cement paste, called admixed corrosion inhibitor (ACI) [11,12], or by applying with brush or spray to the hardened concrete surface diffusing through the pores of the concrete, known as migrating corrosion inhibitor (MCI), see Figure 1 [13,14,15]. Examples of ACIs are amines and fatty acid esters [16], which act through a double mechanism, first, by reducing the ingress of the chlorine ion through the hydrophobic property of the esters and second, by forming a protective layer through the ion-dipole interaction (*δ*^+^_H_ − _N_*δ*^−^) [17].

Sodium nitrite (NaNO_2_) [18], disodium stannate (Na_2_SnO_3_) [19], disodium molybdate (Na_2_MoO_4_), sodium borates (NaBO_2_), trisodium borate (Na_3_BO_3_), cerium nitrate (Ce(NO_3_)_2_) and trisodium phosphate (Na_3_PO_4_) (TSP) have been used as corrosion inhibitors for steel [20,21]. Thermodynamic studies indicate that adsorption of phosphate inhibitor molecules is a spontaneous process governed by physisorption through the Langmuir isotherm model [22,23]. Nitrite-based inhibitors compete with chloride ions in the reaction with ferric iron, favoring the formation of ferric oxide (Fe_2_O_3_) and lepidocrocite (γ-FeOOH) [24]:

2Fe^2+^ + 2OH^−^ + 2NO_2_^−^ → 2NO + Fe_2_O_3_ + H_2_O (1)

Fe^2+^ + OH^−^ + NO_2_^−^ → NO + γ-FeOOH (2)

Because the reactions in Equations (1) and (2) are faster than those related to chloride ions, a stable and protective layer of lepidocrocite is generated. This ability to oxidize ferrous to ferric iron produces more insoluble compounds layers, for instance, *K*_sp_,_Fe(OH)_2__ = 7.9 × 10^−15^, *K*_sp_,_Fe(OH)_3__ = 6.3 × 10^−38^ [25], with a thickness of 17ߝ50 Å [16].

Inorganic corrosion-inhibiting compounds are toxic and have low corrosion-inhibitor efficiency if they are not added in the appropriate amount. In the use of nitrite-type inhibitors, the ratio [NO_2_^−^]/[Cl^−^] needs to be near unity, so that the corrosion inhibition efficiency is maximized [26]. Hybrid inhibitors consisting of inorganic quaternary ammonium salts or phosphates and organic compounds such as imidazole have also been shown to impart high inhibition efficiency [27,28]. Additionally, tertiary amines for the repair of structures have been used as organic corrosion inhibitors [29].

Another method for enhancing the corrosion protection of the embedded rebars is by the use of coatings, as they not only provide a barrier against the electrolyte but also are able to work as a vehicle for inhibitors to further improve the corrosion resistance [30,31,32,33,34]. The use of phosphate chemical conversion (PCC) coatings causes the phosphates to react with Fe ions, forming insoluble compounds, thus impeding the corrosion process [31]. Additionally, phosphates are used as corrosion-inhibitor pigments [35,36,37,38]. Due to its low-cost and low environmental impact, PCC technology has been widely used to improve corrosion resistance by application to the metal surface in order to receive a liquid, powder, or electrodeposited coating [39]. PCC primarily is used to form a layer of iron-zinc phosphate, which is insoluble and corrosion resistant, to produce a protective coating [40]. PCC provides multiple benefits including water resistance and increased adhesion, thus increasing the coating durability. Traditionally, the PCC solution is composed of diluted phosphoric acid (H_3_PO_4_) (PA) and contains metal ions to form coatings, for instance Zn^2+^, Ni^2+^, Fe^2+^ and Mn^2+^ and ions of NO_3_^−^ or NO_2_^−^ as accelerators [39]. Both a hopeite (Zn_3_(PO_4_)_2_∙4H_2_O) coated specimen at pH 3.00 and the scholzite (CaZn_2_(PO_4_)_2_∙2H_2_O) coated specimen at pH 3.75 displayed improved corrosion resistance due to the high ratios of Zn/P and Ca/P, respectively. Alternatively, Ca^2+^ ions have been used as accelerators in the PCC rather than a constituent of the coating [32].

**Figure 2:**
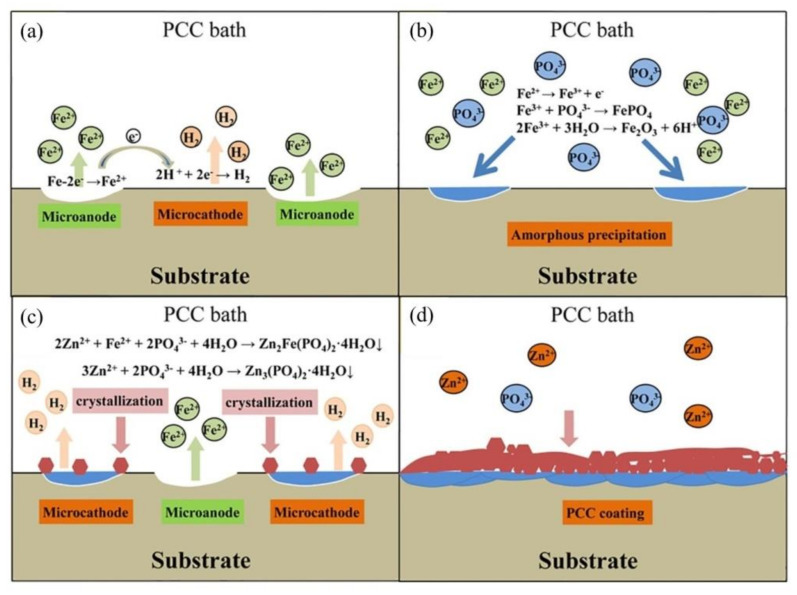
Formation of a phosphate chemical conversion coating: (**a**) microgalvanic couple is established immediately, (**b**) rapid precipitation of amorphous ferric phosphate and ferric oxide on the steel surface, (**c**) insoluble phosphate is deposited and then crystallizes onto the PCC coating on the amorphous base layer, and (**d**) complete PCC coating is formed [33]. Reproduced with permission from Jiang, C. et al., Electrochem. Commun.; published by Elsevier, 2020.

The PCC coating consists of a double layer with an amorphous inner layer and a crystalline phosphate outer layer, according to Jiang et al. [33]. The four stages Jiang et al. proposed are shown in Figure 2; (Figure 2a) the steel dissolution, (Figure 2b) the deposition of the amorphous phase, (Figure 2c) phosphate growth and crystallization and (Figure 2d) the balance of coating dissolution and formation.

**Figure 3:**
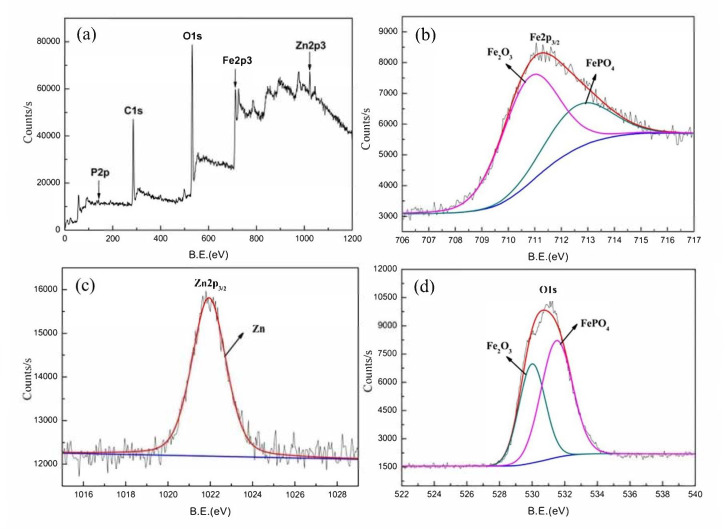
XPS surface analysis: (**a**) the XPS survey spectrum and the XPS spectrum of (**b**) Fe 2p_3/2_, (**c**) Zn 2p_3/2_, and (**d**) O 1 s for samples immersed in the PCC bath for 10 s [33]. Reproduced with permission from Jiang, C. et al., Electrochem. Commun.; published by Elsevier, 2020.

By means of XPS analysis, the formation of the amorphous base layer of Fe_2_O_3_ and FePO_4_ was proven, as seen in Figure 3, where both peaks were recognized in the Fe 2p_3/2_ (Figure 3b) and O 1 s (Figure 3d) spectra. 

The corrosion-inhibition mechanism of phosphates is not fully understood, it is believed that phosphate inhibitors react with the iron ions generated in the corrosion process [41], or with ions present in the mortar, such as calcium, which forms calcium phosphate (Ca_3_(PO_4_)_2_) precipitates, filling the pores and cracks of the mortar, thus impeding the diffusion of aggressive ions [42,43,44,45,46]. It has been found that sodium phosphate (Na_3_PO_4_) can prevent pitting corrosion of steel in the simulated concrete pore solution if its concentration is equal to the chloride concentration [47]. The presence of phosphates in the mortar increases the critical period of pitting initiation from 30 to 100 days and significantly reduces the chloride diffusion rate. Moreover, the apparent chloride diffusion coefficient calculated for mortar containing Na_3_PO_4_·12H_2_O which is around 1.03 × 10^−12^ m^2^/s is lower than that obtained with the reference mortar (2.2 × 10^−12^ m^2^/s) for the same testing period [48].

The concentration of phosphate species inside the pits was higher than the passive film zones without the pit. This indicates that phosphate ions could inhibit the corrosion process through a competitive adsorption mechanism with chloride ions where the chloride attack triggers the phosphate species to further adsorb at the pit locations on the metal surface [49]. In addition, the presence of phosphate ions stabilizes ferrihydrite, a poorly crystallized FeOOH, which may be a protective layer for steel in Cl^−^-contaminated concrete simulating solutions [8]. As a counterpart, the inhibition efficiency of phosphate corrosion inhibitors is decreased in concrete because of the reaction of PO_4_^3−^ ions with the concrete matrix [50].

The surface analysis methods demonstrated that the inhibition mechanism of phosphate ions is attributed to the formation of a passive film with a duplex layer on the metal surface, including the inner layer of iron(hydro)oxides, formed by a solid-state mechanism and the outer layer of iron phosphate complexes mainly as FeHPO_4_, Fe_3_(PO_4_)_2_ and even Fe(PO_4_), formed via a dissolution–precipitation mechanism [49]. 

The impact of phosphate corrosion inhibitors on the stability of the passive film depends on the [Cl^−^]/[OH^−^] ratio. It can be explained by the fact that the high concentration of hydroxyl groups relative to chloride ions causes a predominant effect to form a robust passive film, which in turn stops the pitting corrosion without the aid of phosphate. This corrosion protection mechanism is associated with a continuous increase in the resistance of the passive film by adsorption of phosphate species in the weak points of the passive film, which block the anodic sites [51].

Studies performed under applied mechanical stress revealed that in the presence of phosphate corrosion inhibitors, the critical [Cl^−^]/[OH^−^] ratio increased from 0.4 to 5 for strained electrodes under stress conditions (80% UTS) [52].

The addition of phosphate corrosion inhibitors led to a decrease in chloride binding. This is mainly because phosphates hold a higher priority over chloride during ion exchange in Al_2_O_3_–Fe_2_O_3_-mono (AFm) and –tri (AFt) phases in the Ca–Al–S–O–H system of the concrete matrix. Moreover, phosphates exerted a significant influence on the chemical binding but a negligible effect on the physical binding [53].

More recently, the use of alternative pentasodium triphosphate compounds Na_5_P_3_O_10_, also known as sodium tripolyphospate, has shown an increased inhibition efficiency of around 80% for 480 days exposure in 3.5% NaCl, which is attributed to the development of a protective film barrier of PST on the steel rebar surface. Potentiodynamic polarization results revealed that PST affects the anodic and cathodic sites uniformly, thus presenting a mixed-type corrosion-inhibitor protection mechanism [54]. The use of inorganic corrosion-inhibitor mixtures containing hexametaphosphate compounds (NaPO_3_)_6_, used in 3.5% NaCl contained SCPS, have been proven to reduce the corrosion rate by 8.60 and 25.52 times for 3 and 5% inhibitor addition, respectively [55].

**Figure 4:**
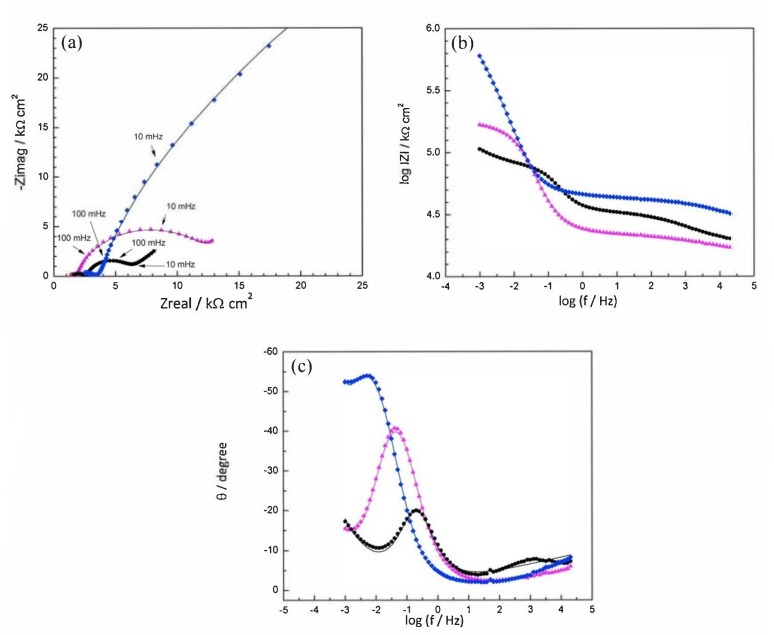
EIS spectra for mix designs: Mix A (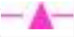), Mix B (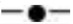) and Mix C (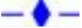), registered after 720 days of exposure. Points represent the experimental EIS data and lines show the fitting results: (**a**) Nyquist plot, (**b**) and (**c**) Bode plots [56]. Reproduced with permission from Yohai, L. et al., Electrochim. Acta; published by Elsevier, 2016.

**Figure 5:**
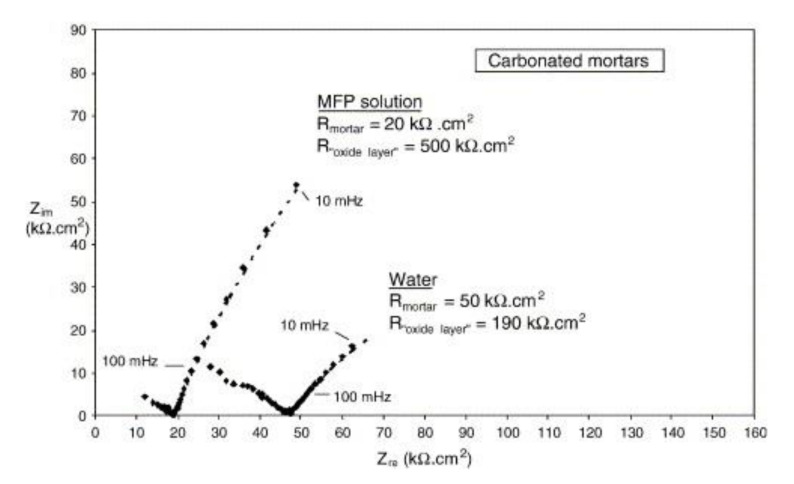
EIS diagrams for carbonated mortars at 48 h after application of aqueous solutions (MFP or water) [61]. Reproduced with permission from Chaussadent, T. et al., Cem. Conc. Res.; published by Elsevier, 2006.

**Figure 6:**
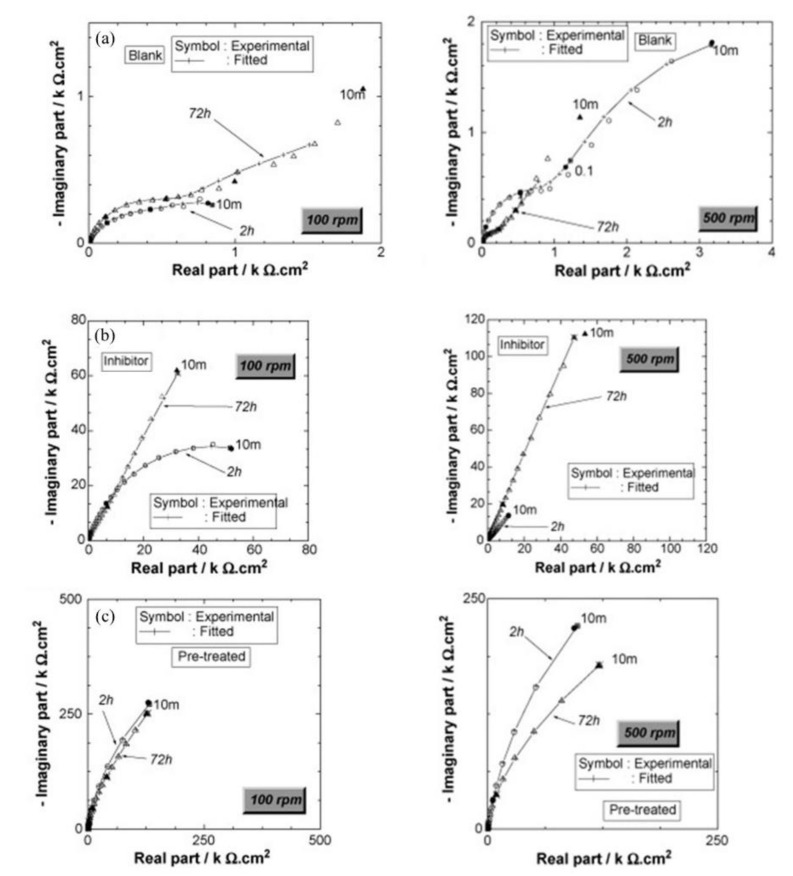
Impedance diagrams (Nyquist representation) of (**a**) steel electrode/S_1_ solution, (**b**) steel electrode/S_2_ solution and (**c**) pretreated steel electrode/S_1_ solution; for two periods of immersion: 2 and 72 h and for two electrode rotation rate values: 100 and 500 rpm [45]. Reproduced with permission from Etteyeb, N. et al., Electrochim. Acta; published by Elsevier, 2007.

Disodium hydrogen phosphate (Na_2_HPO_4_, DHP) in simulated concrete pore solution (SCPS) and in mortar acts as an anodic corrosion inhibitor [43,46]. Overall, phosphates require oxygen to be effective, as they are nonoxidizing anodic inhibitors [38]. Trisodium phosphate (Na_3_PO_4_∙H_2_O, TSP) in mortar acts as a mixed corrosion inhibitor [56], as well as in chloride environments containing a [PO_4_^3−^]/[Cl^−^] ratio higher than 0.6 [41,57] and even as a cathodic corrosion inhibitor, with a [PO_4_^3−^]/[Cl^−^] ratio less than 0.6 [58]. Impedance techniques, such as electrochemical impedance spectroscopy (EIS), can be used to measure the corrosion improvement of inhibitors [30,45,56,59,60]. Yohai et al. showed in their work with mortars great corrosion enhancement of the TSP (Mix C), even with the chloride addition, over the blank (Mix A) and the chloride contaminated specimen (Mix B) (see Figure 4a), clearly seen in the Bode plot where Mix C is of one order of magnitude higher than the other two mixes (see Figure 4b) [56]. Similarly, Chaussadent et al. found an improvement in the corrosion protection by sodium monofluorophosphate as a corrosion inhibitor using the EIS results of mortar samples after carbonation (Figure 5) [61]. Another example of the use of EIS is the work from Etteyeb et al., in which the specimen containing inhibitors (see Figure 6a) increased its impedance by two orders of magnitude compared to the blank (see Figure 6b) [45]. 

**Figure 7:**
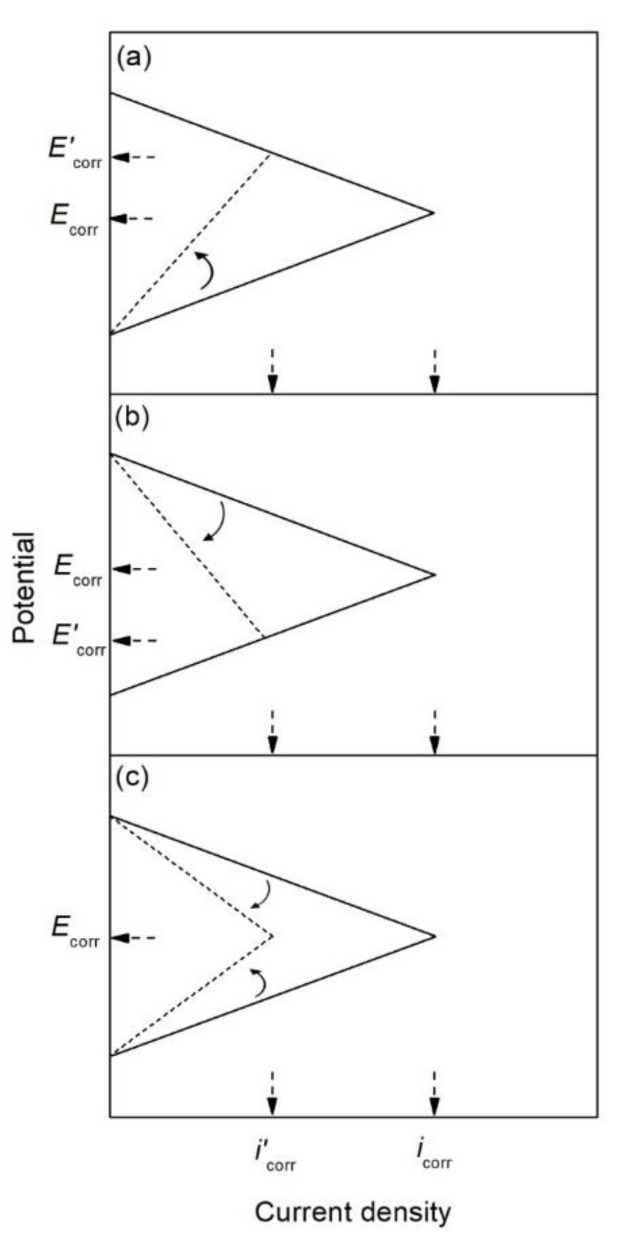
Evans diagrams showing the effect of a corrosion inhibitor, (**a**) on the anodic branch (anodic inhibitor), (**b**) on the cathodic branch (cathodic inhibitor), and (**c**) on both the anodic and cathodic branches (mixed inhibitor).

**Figure 8:**
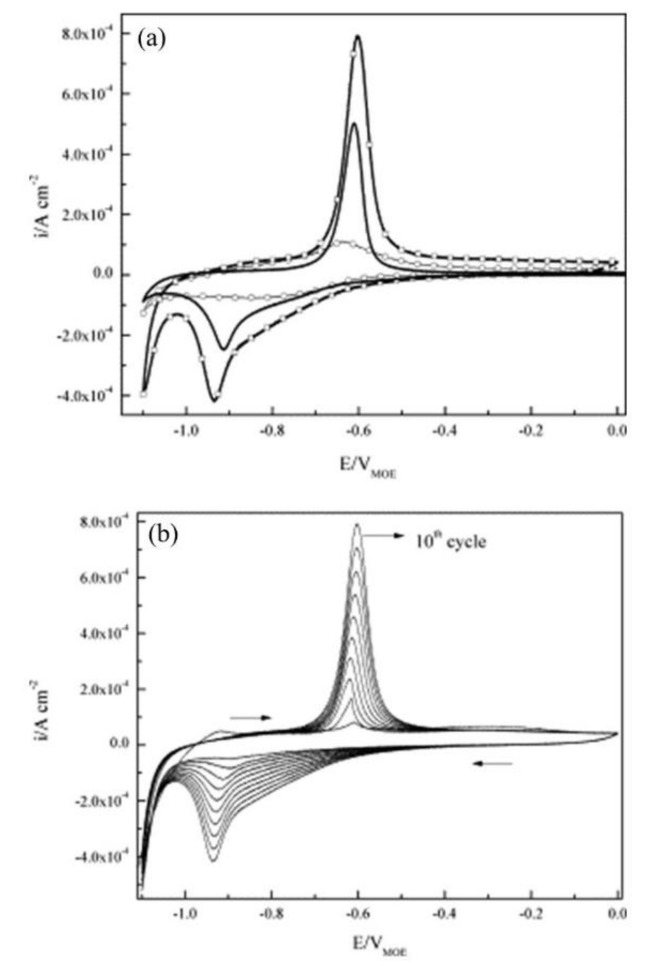
Cyclic voltammograms for steel: (**a**) (tenth cycle) in PSS (—), PSS + Cl^−^ (–□–), PSS + Cl^−^ + PO_4_^3−^ (–○–), and (**b**) cycles 1–10th in PSS + Cl^−^. Scan rate: 10 mV s^−1^ [60]. Reproduced with permission from Yohai, L. et al., Electrochim. Acta; Published by Elsevier, 2013.

**Figure 9:**
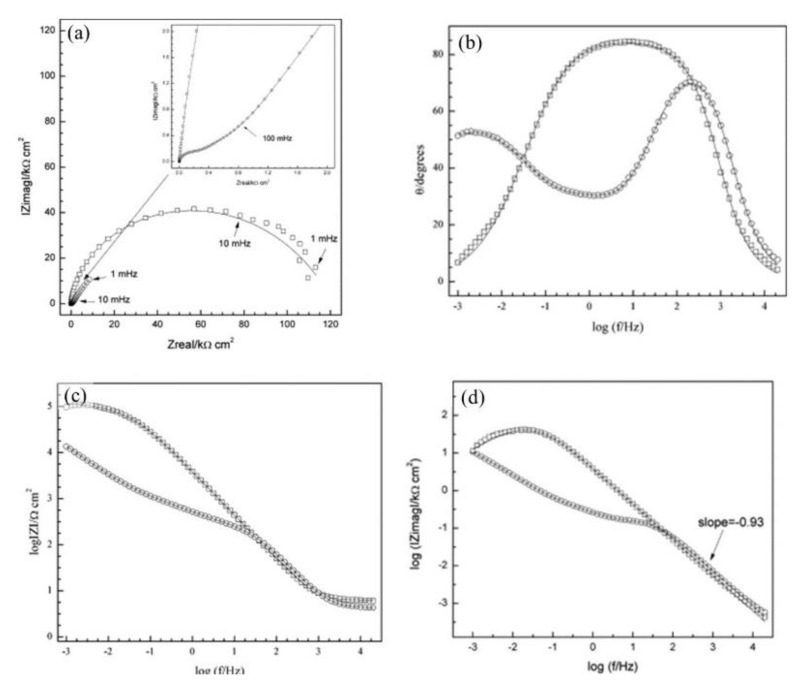
Impedance spectra recorded on steel electrodes aged over 24 h at *E*_corr_ in SSP + Cl^−^ with and without inhibitor. The symbols represent the data and the lines the fitting results. (**a**) Nyquist representation, (**b**) and (**c**) Bode representation, and (**d**) imaginary part of impedance as function of frequency, in logarithmic scale. PSS + Cl^−^ (–□–), PSS + Cl^−^ + PO_4_^3−^ (–○–) [60]. Reproduced with permission from Yohai, L. et al., Electrochim. Acta; Published by Elsevier, 2013.

The corrosion inhibitor effect on the anodic and cathodic polarization curves can be seen in the Evans diagrams shown in Figure 7. This is in agreement with the findings of Yohai et al., in which [PO_4_^3−^]/[Cl^−^] = 1 and phosphate behaves as a mixed inhibitor [60] and phosphate ions promote ferrous phosphate precipitation due to the higher solubility of ferric phosphate (p*K*_sp_ = 26) than ferrous phosphate (p*K*_sp_ = 32). The cyclic voltammograms in PSS, PSS + Cl^−^ and PSS + Cl^−^ + PO_4_^3−^ showed the presence of a single negative and positive peak, attributed to the accumulation of magnetite on the steel surface, which is not fully reduced (see Figure 8a) [60]. However, for the PO_4_^3−^ ions, which promote Fe_3_(PO_4_)_2_ precipitation, the difference was not substantial and is due to lack of formation of Fe^3+^ compound, hence not getting reduced in the following cycles (see Figure 8b). By the impedance fitting, the protectiveness of the PO_4_^3−^ ions was also seen, showing more ideal capacitors, which were related to the presence of a protective passive layer, however a small decrease in the impedance was associated with the change in the film composition, influencing the electronic properties (see Figure 9).

It has been also observed that TSP behaves as an anodic corrosion inhibitor, which penetrates through the pores and necessitates the use of high concentrations [45,62], which can cause rheological changes to the mortar. TSP increases the critical concentration threshold of the ratio [Cl^−^]/[OH^−^] for steel in SCPS and in mortar [63]. Corrosion begins in active sites of the reinforcement when the concentration of chloride at the steel/concrete interface exceeds a critical value [64]. The corrosion rates for various measurements are presented in Table 1 as an example [65].

Table 2 shows corrosion potential (*E*_corr_) and corrosion current density (*i*_corr_) measured for various phosphates [8,15,38,61,63,64,65,66]. The probability for active corrosion, according to ASTM C876 is high (~90%) for *E*_corr_ < −0.27 V vs. SCE, uncertain for −0.27 V < *E*_corr_ < −0.12 V vs. SCE and 10% chance for corrosion for *E*_corr_ > −0.12 V vs. SCE [67]. 

Calcium monofluorophosphate (CaPO_3_F) [68], as well as zinc monofluorophosphate (ZnPO_3_F) have been used as corrosion inhibitors for steel in 3 wt.% NaCl solution [69], in both cases, the inhibitors are shown to be effective. Manganese monofluorophosphate (MnPO_3_F) has been found to exhibit a mixed corrosion inhibition for steel in 3 wt.% NaCl solution [70]. Aluminum tri-polyphosphate (AlH_2_P_3_O_10_∙2H_2_O) has been used as an ACI inhibitor with good results [71].

**Figure 10:**
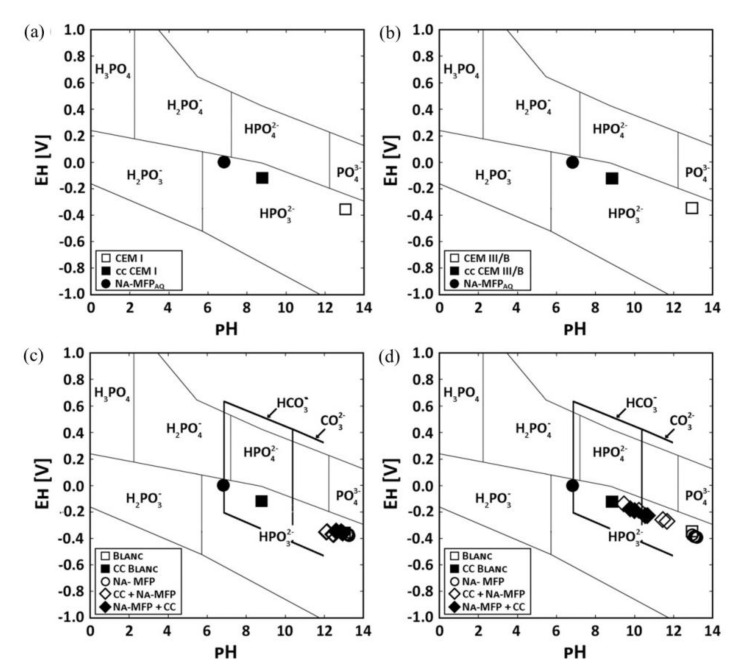
Pourbaix diagram of two different OPC cement pastes (CEM I and CEM III/B) showing the stability fields for phosphoric ions in solution and the *E*_H_−pH values of the pore solutions. pH values of the pore solutions from untreated CEM I, carbonated paste (cc) and the self-healing agent sodium monofluorophosphate (MFA) for: (**a**) CEM I and (**b**) CEM III/B, respectively. pH values of the pore solutions from paste samples that were either only impregnated with MFA or were first carbonated and impregnated with MFA (cc + MFA) or were impregnated with MFA and carbonated (MFA + cc) for: (**c**) CEM I and (**d**) CEM III/B, respectively. The thick black lines frame the stability of HCO_3_^−^ and CO_3_^2−^ [72]. Reproduced with permission from Kempl, J. et al., Cem. Conc. Comp.; Published by Elsevier, 2016.

Figure 10 shows Pourbaix diagrams with the stability fields for phosphoric ions as well as *E*_H_−pH values of pore solution of two OPC pastes labelled CEM I and CEM III/B [72]. The use of sodium monofluorophosphate (Na_2_PO_3_F, MFP) dual inhibitor and self-healing agent was found to recover 98.85% and 79.82% of the pH of the carbonated cement pastes compared to the untreated paste for CEM I and CEM III/B, respectively. The pH increased for higher concentrations of sodium in the treating agent. 

Natural, organic-based corrosion-inhibitor products have been used as an alternative to inorganic compounds because they are nontoxic, have a low environmental impact, are biodegradable, provide good corrosion inhibition efficiency and are low cost. The inhibition of these compounds depends on the molecular structure and the affinity and compatibility with steel [73,74,75,76]. Organic MCIs diffuse to the anodic or cathodic sites and adsorb on the steel surface through covalent bonds and polar groups [77,78,79].

Amines, aliphatic carboxylic acids and saturated fatty acids have been used as ACIs for steel embedded in concrete [80]. Benzoate and its amino derivatives and dicarboxylates [81], as well as carboxylic acids have been used as organic ACIs as well [82]. Heterocyclic compounds are widely used, the heteroatoms of the imidazole compounds contribute the lone pair electrons to the vacant iron orbital [83,84]. The curing process of OPC can be affected by the air entrainment of organic compounds [85]. Thus, the incorporation of organic ACIs causes a decrease in the compressive strength of the concrete (~15%) [86].

Surfactants are frequently used in concrete, they are organic molecules formed by a polar hydrophilic group (head) attached to a nonpolar hydrophobic group (tail), this unique chemical architecture leads to a broad spectrum of self-assembly phenomena. The surfactant corrosion inhibitors have advantages, such as high inhibition efficiency, low toxicity, low cost and availability [34,87]. When the concentration of the adsorbed surfactant on the metal surface is high enough (micelles), a bilayer, or a multilayer, forms, which seals the metal surface and prevents corrosion [88]. For this type of inhibitor, the critical concentration of the micelle (CCM) is the most important parameter. The adsorption of an inhibitor on the metal surface depends mainly on the charge of the metal, the dipole moment of the inhibitor and the adsorption of other ionic species on the metal. The potential of zero charge (E_PZC_) plays a decisive role in the electrostatic adsorption process [89]. The metal charge (*ϕ*) is calculated as *ϕ* = *E*_corr_ – *E*_PZC_ [90].

The use of nano/microcapsules to store an inhibitor prevents premature leaching of the active substances and reduces the loss of effectiveness [91]. Based on the concept of chemical self-healing, sodium citrate (C_6_H_5_O_7_Na_3_∙2H_2_O) [77,92] and MFP encapsulated in ethylcellulose has been used as an ACI [3]. Zeolites have been used as agents to encapsulate organic inhibitors [93]. The encapsulation of an inhibitor implies that the release of the active substance only takes place under the presence of aggressive agents and in conditions in which the steel is prone to corrosion [91,94,95].

The use of biological inhibitors is based on biomolecules such as those derived from green plants that are used as ACIs and, also, are used by applying the inhibitor to concrete, acting as MCIs [96,97]. Other inhibitors are made up of yeast extracts, bacterial cells or other organic substances [98]. Biosurfactants emitted by bacterial cells are potentially interesting as ecological mixtures, because they contain in the same molecule parts that act as hydrophilic (head) and parts that act as hydrophobic (tail). Using bacterial cell biosurfactants, the hydrophobic parts can be saturated/unsaturated hydrocarbon chains or fatty acids, while the hydrophilic parts are formed by an acid, peptide or mono-, di- or polysaccharides [99].

This critical review aims to study the corrosion-inhibition mechanism of soluble phosphates for steel embedded in OPC mortar in the presence of 3.5 wt.% NaCl solution. Special attention was given to MFP, DHP and TSP as conventional inhibitors for steel reinforcement repair. The effectiveness of the corrosion inhibitors was evaluated, for the MCI method through the immersion of mortar specimens in an aqueous phosphate solution, as well for the ACI method through addition to the OPC as an admixture.

## 2. Description of Phosphate Behavior, Methodology

Three phosphate compounds, sodium monofluorophosphate (MFP), disodium hydrogen phosphate (DHP) and trisodium phosphate (TSP) were studied as corrosion inhibitors of steel reinforcements embedded in OPC mortar, using two water/cement (w/c) ratios, 0.5 and 0.6, in 3.5 wt.% NaCl solution. The corrosion inhibitors were deployed in two different ways, by immersion of OPC specimens after the curing period in aqueous solution containing the soluble phosphates (MCI) or by addition of the phosphate powders to a fresh OPC paste (ACI). Carbon steel rebar of 8 mm in diameter and a chemical composition of 0.45% C, 0.22% Si, 0.72% Mn, <0.010% P, 0.022% S, 0.13% Cr, 0.13% Ni, 0.18% Cu. and the balance Fe, were used as reinforcements. Type I 52.5 N/SR OPC with the chemical composition given in Table 3 was used. The specimens were studied using electrochemical DC techniques, where corrosion potential (*E*_corr_) was monitored and polarization resistance (*R*_p_) was obtained by linear polarization resistance (LPR) measurements for 70 days. A three-electrode cell configuration was used for electrochemical testing, using the rebar as the working-electrode, 1 cm^2^ surface area, a platinum mesh as counter-electrode and a saturated calomel reference electrode (SCE). The *E*_corr_ was monitored until a stable value was observed. LPR tests were recorded by applying a ±20 mV vs. *E*_corr_. All tests were done in triplicate.

## 3. Effect of Phosphate on the Mortar Matrix

The electron probe micro-analysis (EPMA) technique was used to determine the phosphorous content (% P_2_O_5_) versus penetration depth for the MCI specimens [43]. The P_2_O_5_ content averaged near 0.10% after an initial sharp decrease. The intercept of the tangent from the sharp decay of the curve denotes the P_2_O_5_ penetration depth. The compounds DHP and TSP displayed penetration values of 380 μm and 126 μm, respectively, whereas MFP measured a penetration depth of 1114 µm. 

**Figure 11:**
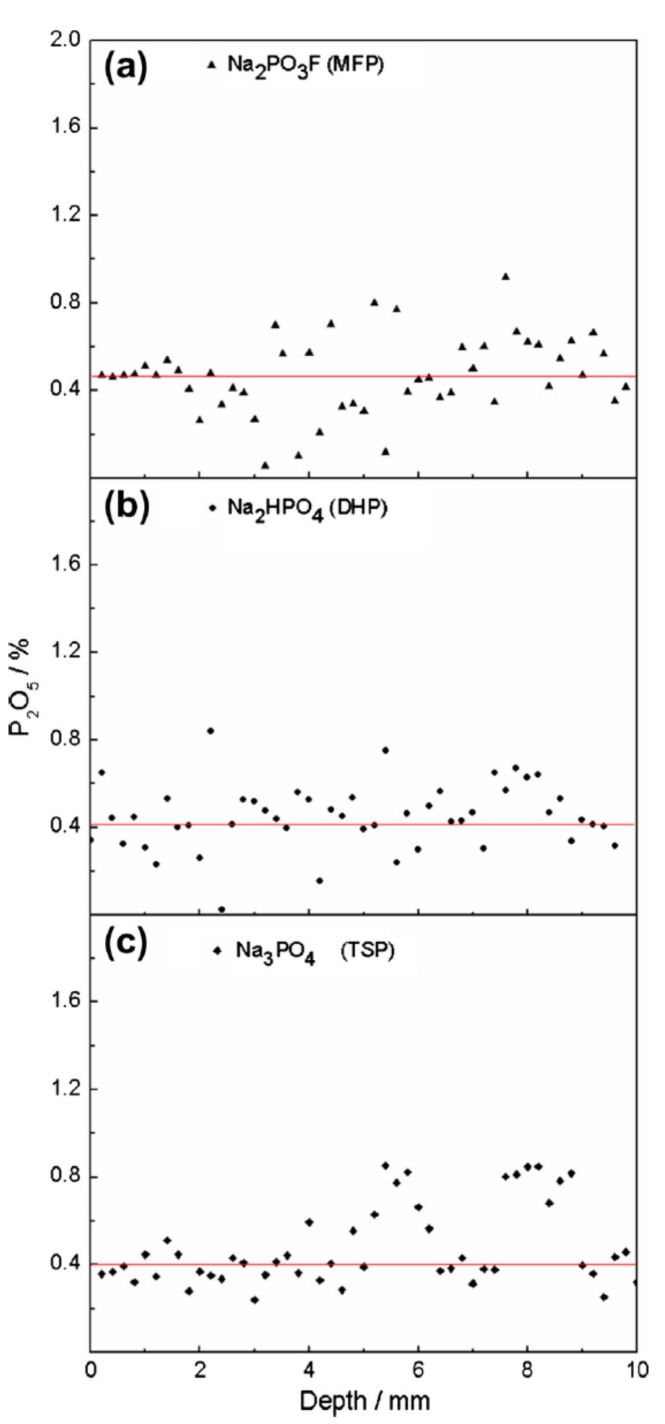
Content of P_2_O_5_ versus penetration depth in the presence of admixture corrosion inhibitor (ACI): (**a**) MFP, (**b**) DHP and (**c**) TSP [43]. Reproduced with permission from Bastidas, D.M. et al., Cem. Conc. Comp.; published by Elsevier, 2013.

**Figure 12:**
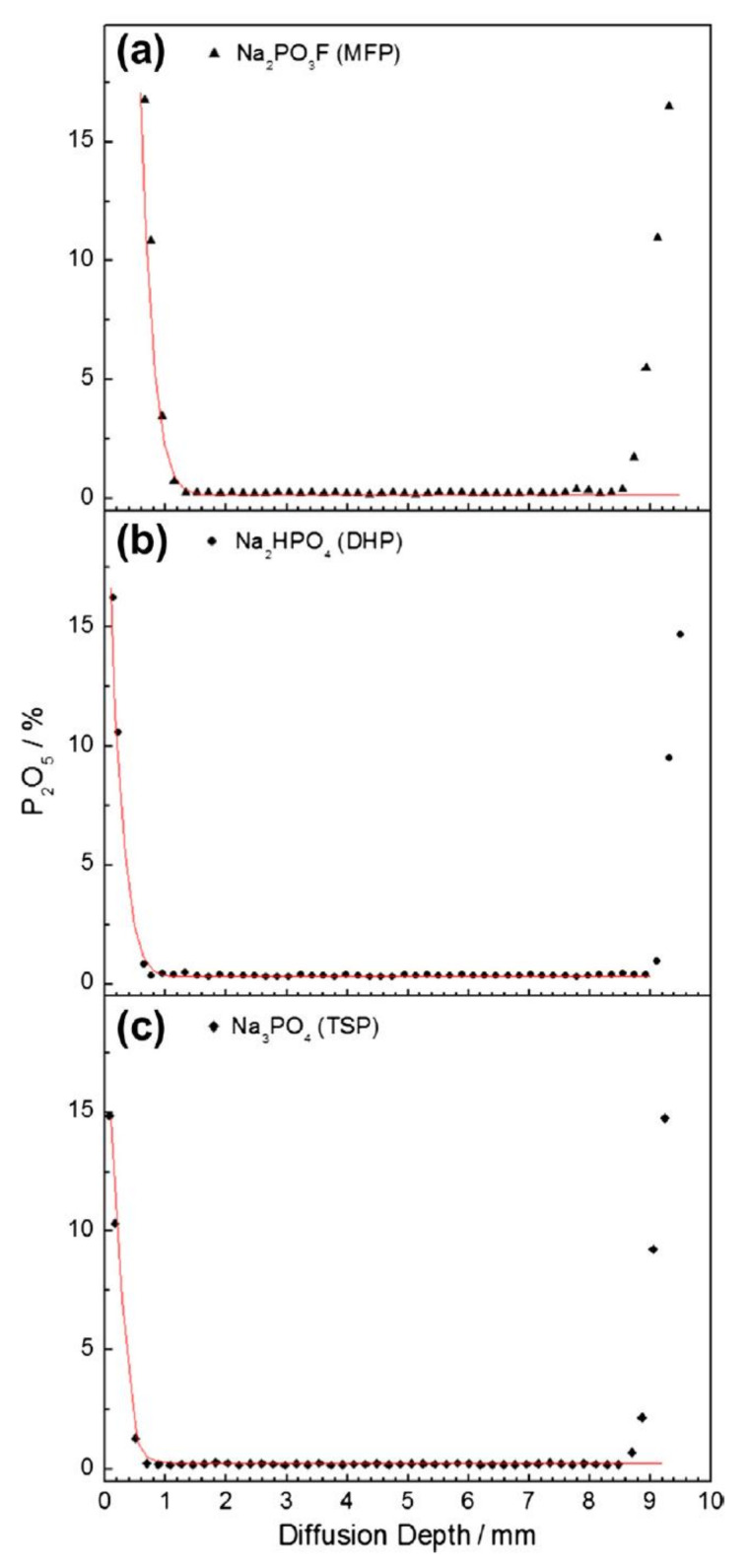
Content of P_2_O_5_ versus penetration depth in the presence of migrating corrosion inhibitor (MCI): (**a**) MFP, (**b**) DHP and (**c**) TSP [43]. Reproduced with permission from Bastidas, D.M. et al., Cem. Conc. Comp.; published by Elsevier, 2013.

The phosphorous content as a function of penetration depth for ACI specimens using the EPMA technique is shown in Figure 11 and displays values between ~0.10–0.90% [43]. The MFP, DHP and TSP displayed an average P_2_O_5_ content of 0.47%, 0.45% and 0.43%, respectively. It was determined that the OPC paste with an ACI specimen with 0.43–0.47% P_2_O_5_ (Figure 11) contained higher amounts of phosphate compared to the MCI specimen with ~0.10% P_2_O_5_ content (Figure 12) due to the 190 μm distance between measurements, spot diameter of 20 μm and that no analysis had a P_2_O_5_ value of less than 0.10%.

**Figure 13:**
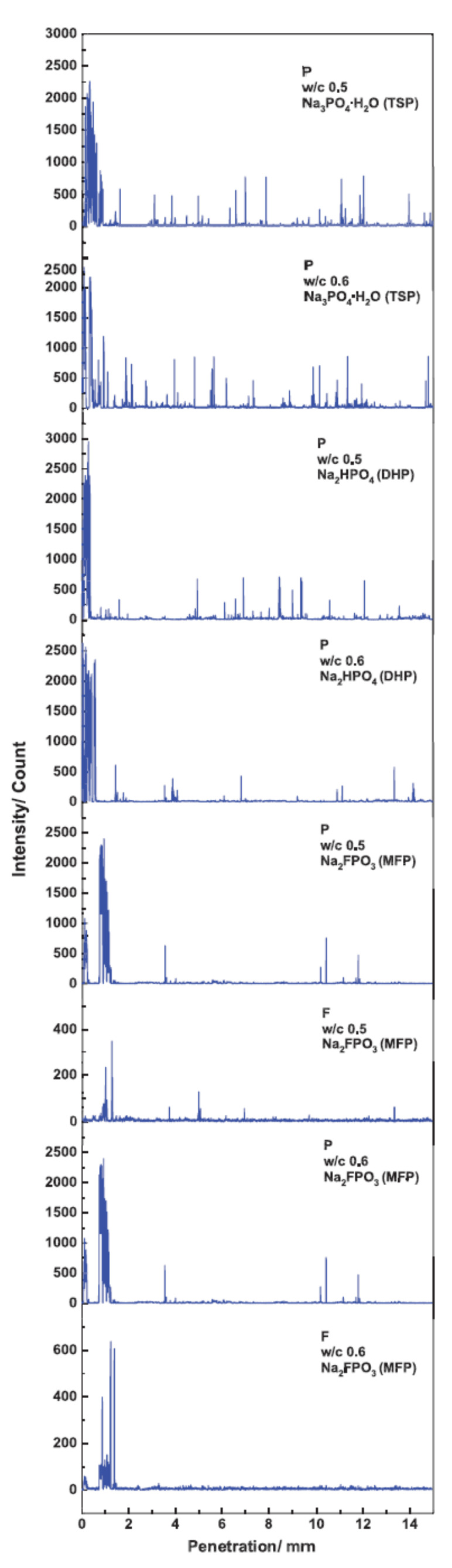
Electron probe micro-analysis (EPMA) of intensity versus penetration depth [44]. Reproduced with permission from Criado, M. et al., Eur. J. Environ. Civ. Eng.; published by Taylor and Francis, 2017.

The six mortar specimens in 5 wt.% TSP, 5 wt.% DHP or 5 wt.% MFP aqueous solution at room temperature were analyzed by EPMA for P and F, shown in the line profile in Figure 13 [44]. A trend can be observed in all the tested specimens, displaying a penetration depth of 2 mm maximum, subsequently, the P and F content decreased to near zero. The mortar specimens with w/c ratios of 0.5 and 06 immersed in 5 wt.% TSP solution showed the greatest amount of low intensity peaks. However, virtually no high peaks were observed for either specimen with w/c ratio of 0.6 in 5 wt.% DHP solution or the specimen with w/c ratio of 0.5 or 0.6 in MFP solution, thus demonstrating low penetration. The penetration depths are listed in Table 4 for each specimen, the greatest depth was that of the MFP compound (1.40 mm) followed by TSP and DHP.

It should be noted that differences were not observed in the penetration of MFP, DHP or TSP for the two w/c ratios (0.5 and 0.6), which may be interpreted that they are too close to observe any effect. It has been reported that MFP imparts effective corrosion-inhibition protection only for reinforcing steel bars with concrete cover not thicker than 1 cm [100].

**Figure 14:**
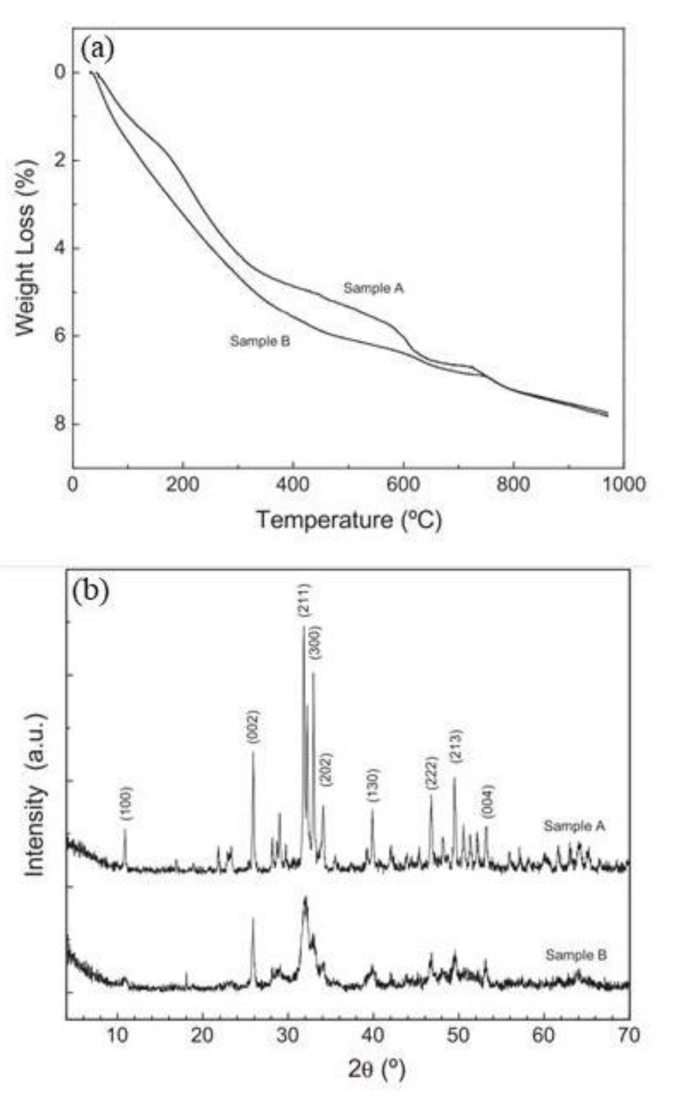
Phosphate samples obtained using 0.4 M NaHPO_4_ and 500 mL of 0.6 M Ca(NO_3_)_2_·4H_2_O at pH ~12.5 (sample A) and pH ~8.5 (sample B): (**a**) thermogravimetric (TGA) results, and (**b**) X-ray diffraction (XRD) patterns for samples A and B [42]. Reproduced with permission from Bastidas, D.M. et al., Constr. Build. Mater.; published by Elsevier, 2010.

The thermogravimetric analysis (TGA) for samples A and B is shown in Figure 14 [42]. Sample A was fabricated using 500 mL of 0.4 M NaHPO_4_ and 500 mL of 0.6 M Ca(NO_3_)_2_·4H_2_O and at pH ~12.5. Sample B was prepared using 500 mL of 0.4 M NaHPO_4_ and 500 mL of 0.6 M Ca(NO_3_)_2_·4H_2_O and at pH ~8.5. Sample A showed continuous weight loss (4.50% total) within the range of 50–400 °C, likely caused by occluded water evaporation. Within the ranges of 550–630 °C and 700–800 °C were two weight reductions, likely caused by the dehydroxylation of the hydroxyapatite (Ca_5_(PO_4_)_3_(OH)) (HAP). Sample B showed a similar behavior to sample A. A gradual weight reduction of 6.50% was observed over the range of 25–600 °C, signifying a low crystallinity. Again, two decreasing steps are shown, attributed to the dehydroxylation of the HAP in the ranges of 600–630 °C and 700–800 °C, corroborating the XRD results (Figure 14b).

## 4. Effect of Phosphate on the Steel Reinforcement 

**Figure 15:**
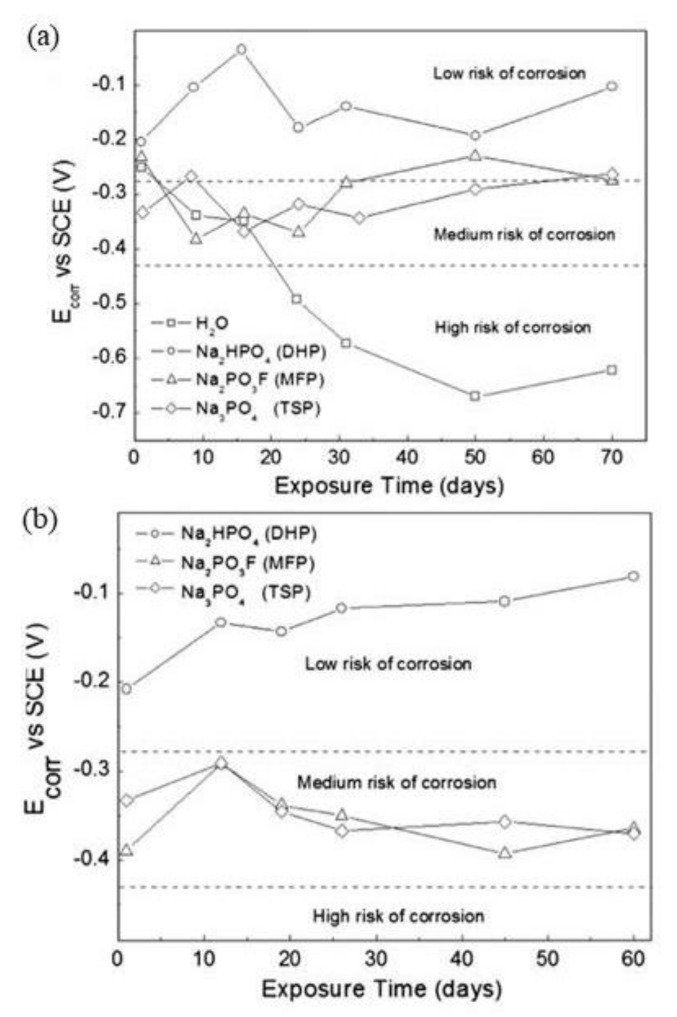
Corrosion potential (*E*_corr_) versus time for steel in the presence of: (**a**) migrating corrosion inhibitor (MCI) and (**b**) admixture corrosion inhibitor (ACI) [43]. Reproduced with permission from Bastidas, D.M. et al., Cem. Conc. Comp.; published by Elsevier, 2013.

MCI specimens with embedded steel rebar in mortar and immersed in 0.2 M phosphate solutions (MFP, DHP, TSP) or distilled water for the control specimen were monitored for *E*_corr_ versus time and are shown in Figure 15, top [43]. Overall, the *E*_corr_ values were shown to be within the level of low or uncertain risk for corrosion, for *E*_corr_ < −0.28 V vs. SCE the probability of corrosion was high (>90%); for −0.28 V< *E*_corr_ < −0.12 V vs. SCE corrosion was uncertain; and for *E*_corr_ > −0.12 V vs. SCE there was a 10% probability of corrosion [67]. The control test alone measured *E*_corr_ values that corresponded to a high risk of corrosion and the results from MFP, DHP and TSP suggest they acted as anodic inhibitors. The ACI specimens are shown in Figure 15a for embedded steel rebar in mortar with a blending of 3 wt.% MFP, DHP, or TSP solid powders with OPC, water and sand. The ACI specimens were then stored in desiccators at ~95% relative humidity (RH). The DHP showed the best corrosion inhibitor performance.

**Figure 16:**
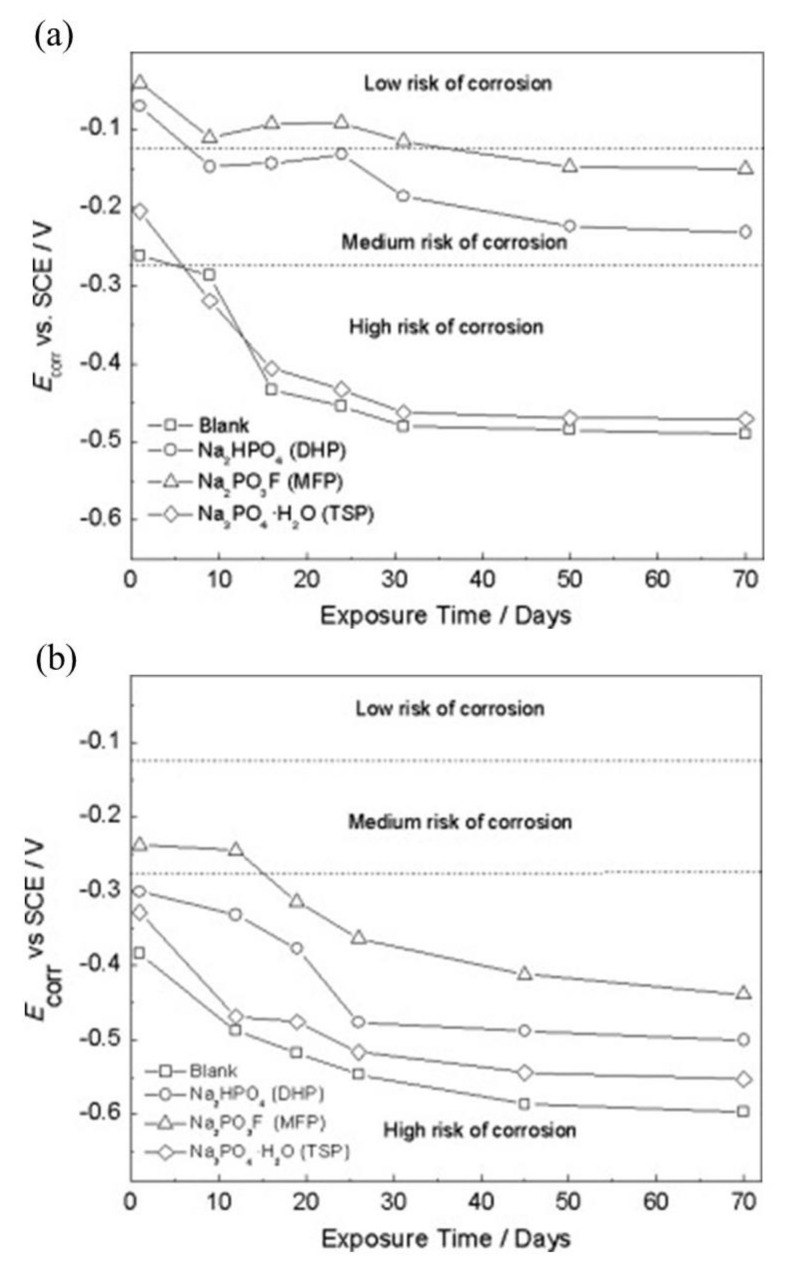
Corrosion potential (*E*_corr_) versus time for steel in the presence of: (**a**) migrating corrosion inhibitor (MCI) and (**b**) admixture corrosion inhibitor (ACI) bottom and in contact with 3.5 wt.% NaCl [46]. Reproduced with permission from Bastidas, D.M. et al., Cem. Conc. Comp.; published by Elsevier, 2015.

The *E*_corr_ versus time plot for MCI specimens with embedded steel rebar in mortar with 0.2 M phosphate solutions (MFP, DHP or TSP) contaminated with 3.5 wt.% NaCl is shown in Figure 16a [46]. The *E*_corr_ values displayed corrosion risk levels of low, uncertain and high [67]. The MCI specimens containing MFP, DHP, TSP inhibitors showed less negative *E*_corr_ values compared to the control specimen, with the exception of the TSP solution, which offered a high risk of corrosion. The *E*_corr_ values over time for the ACI specimens with embedded rebar in mortar blended with 3 wt.% solid powders of MFP, DHP, or TSP with OPC, water and sand and contaminated with 3.5 wt.% NaCl are shown in Figure 16a. Similar to the MCI specimens, the best corrosion inhibitor observed for the ACI specimens was the MFP with a medium and high risk of corrosion according to the *E*_corr_ values. Therefore, the MFP as well as the DHP and TSP compounds may be considered to be anodic inhibitors. 

**Figure 17:**
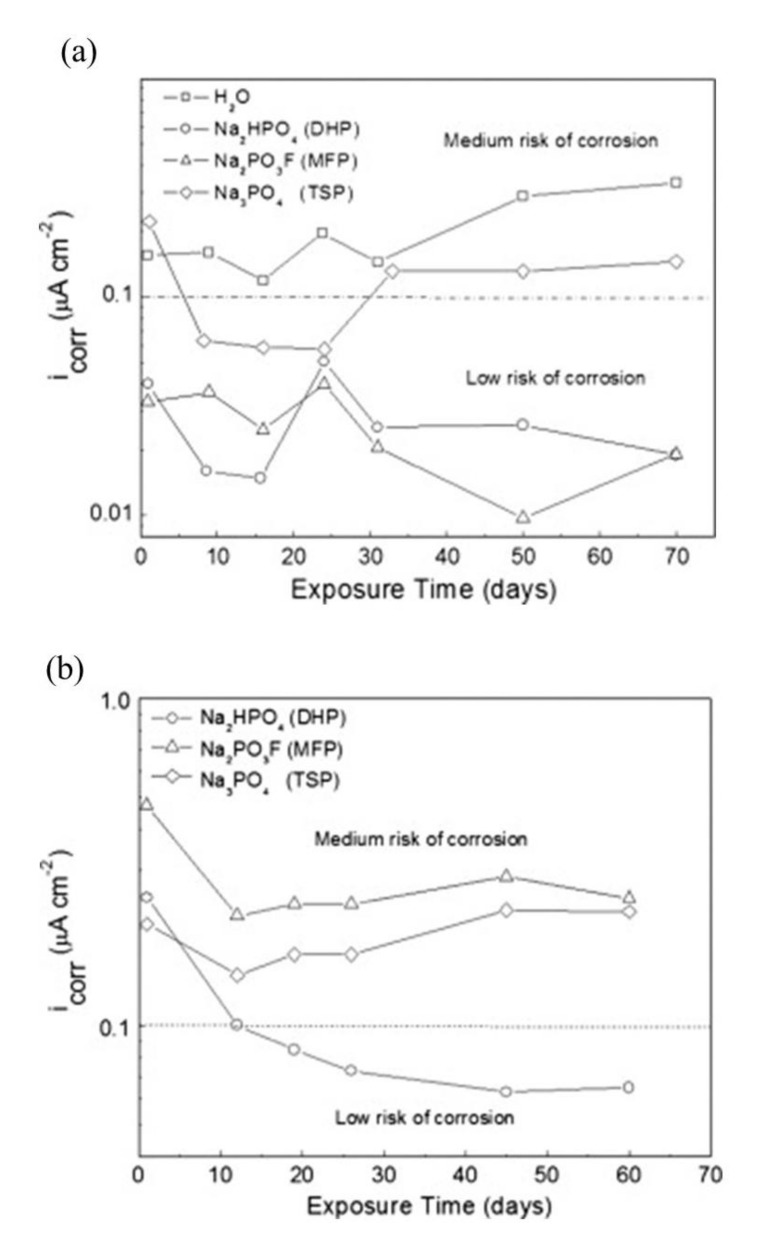
Corrosion current density (*i*_corr_) versus time for steel in the presence of: (**a**) migrating corrosion inhibitor (MCI) and (**b**) admixture corrosion inhibitor (ACI) bottom [43]. Reproduced with permission from Bastidas, D.M. et al., Cem. Conc. Comp.; published by Elsevier, 2013.

The corrosion current density (*i*_corr_) measured for the MCI specimens over the test period is shown in Figure 17a. The specimens contained steel rebar embedded in mortar submerged in 0.2 M MFP, DHP, or TSP solutions, with a distilled water control specimen [43]. The measured *i*_corr_ values depict the samples within low or medium risk for corrosion [65]. The dotted line located at 0.1 µA/cm^2^ depicts the estimated limit for steel passivity [65,101]. The MFP and DHP compounds showed the best inhibitive performance for the MCI specimens measuring *i*_corr_ values below 0.1 µA/cm^2^. Between 10 and 30 days, the TSP showed passivity, however, the *i*_corr_ subsequently increased. The control sample showed active corrosion. The *i*_corr_ values for the ACI specimens prepared with embedded steel rebar with a blend of 3 wt.% MFP, DHP, or TSP solid powders with OPC, water and sand are shown in Figure 17a. The ACI specimens were stored in desiccators at ~100% HR. The DHP compound was shown to be the best corrosion inhibitor for ACI specimens, showing *i*_corr_ values at low or medium risk levels for corrosion. 

**Figure 18:**
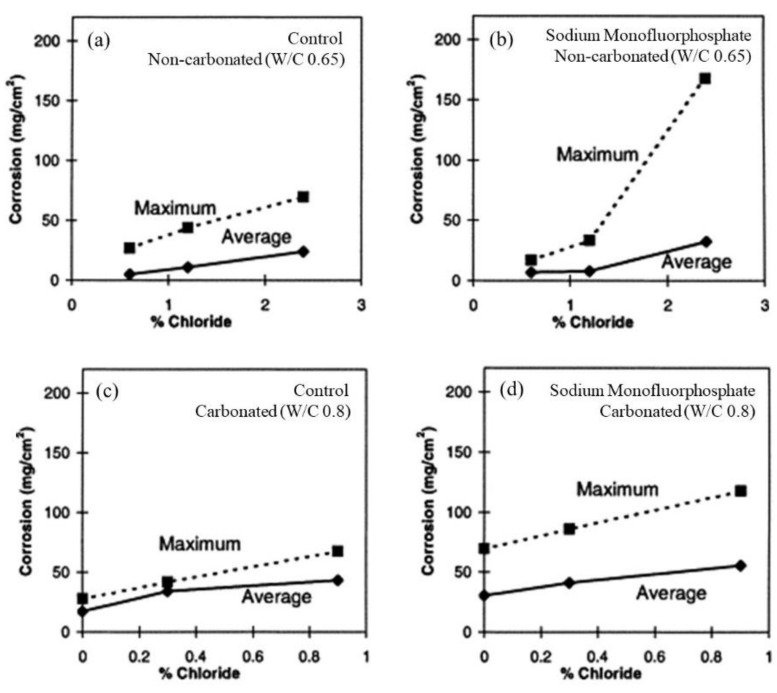
Average and maximum corrosion weight losses for steel bars at three cover depths in various concrete specimens: (**a**) control noncarbonated, (**b**) sodium monofluorophosphate noncarbonated, (**c**) control carbonated, and (**d**) sodium monofluorophosphate carbonated [15]. Reproduced with permission from Ngala, V. et al., Corros. Sci.; published by Elsevier, 2003.

Figure 18 shows the average and maximum corrosion weight losses for steel bars embedded in concrete for noncarbonated and carbonated conditions [15]. The measured weight losses showed a scarce reduction for Na_2_PO_3_F^−^ treated noncarbonated specimens, while a minor increase was seen in the carbonated samples, related to the hydrolysis of PO_3_F^−^ promoting the formation of F^−^ ions.

**Figure 19:**
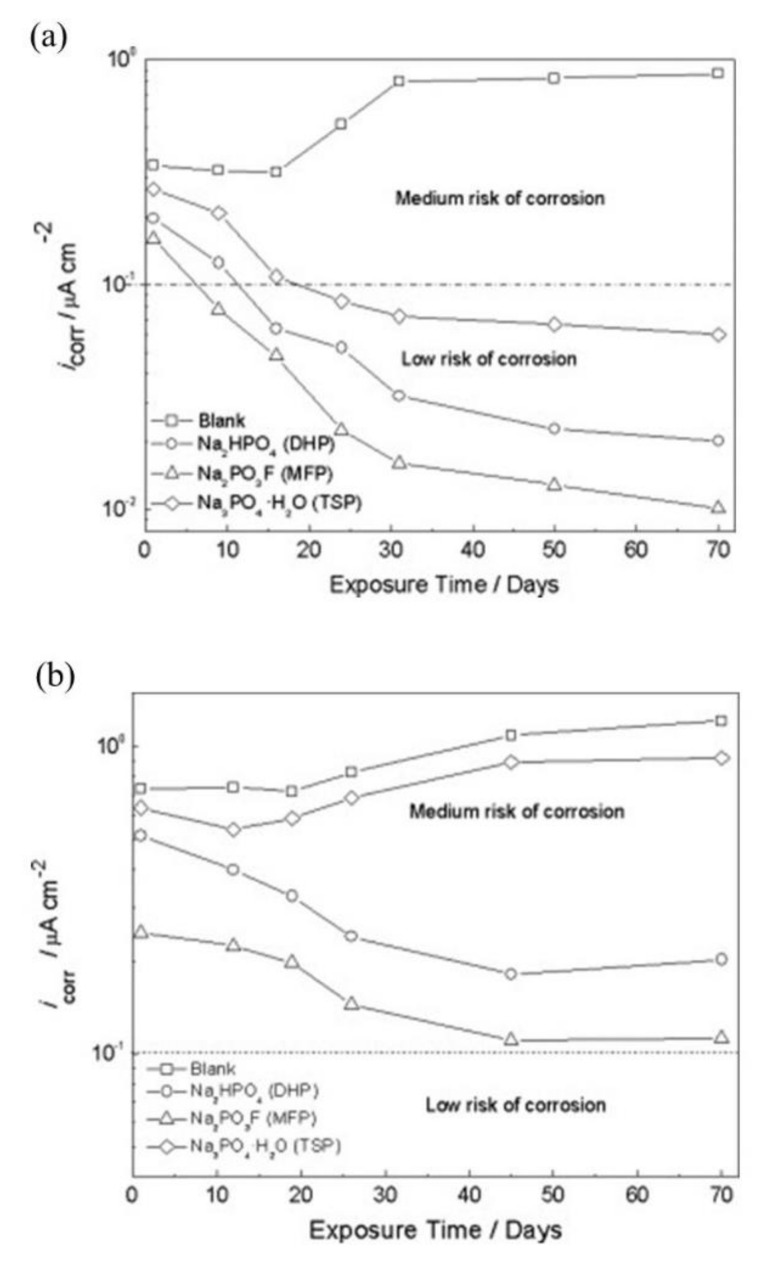
Corrosion current density (*i*_corr_) versus time for steel in 3.5 wt.% NaCl in the presence of: (**a**) migrating corrosion inhibitor (MCI), and (**b**) admixture corrosion inhibitor (ACI) [46]. Reproduced with permission from Bastidas, D.M. et al., Cem. Conc. Comp.; published by Elsevier, 2015.

The *i*_corr_ values for the MCI specimens with steel rebar embedded in mortar in 0.2 M MFP, DHP, or TSP solution with 3.5 wt.% NaCl contamination are shown in Figure 19a [46]. According to the *i*_corr_ values, the specimens are within low to medium corrosion risk [67]. MFP and DHP showed the best corrosion inhibition for the MCI specimens with *i*_corr_ values below <0.1 µA/cm^2^. The control sample showed active corrosion over the test period. The *i*_corr_ values for the ACI specimens with embedded steel rebar prepared by blending 3 wt.% MFP, DHP, or TSP and OPC, water, sand and 3.5 wt.% NaCl contamination are shown in Figure 19a. The MFP compound showed the highest corrosion inhibitor performance and had *i*_corr_ values between low or medium corrosion risk. The DHP and TSP compounds also displayed medium corrosion risk, while the control specimens had *i*_corr_ between 0.3–0.9 µA/cm^2^, close to the TSP values. 

**Figure 20:**
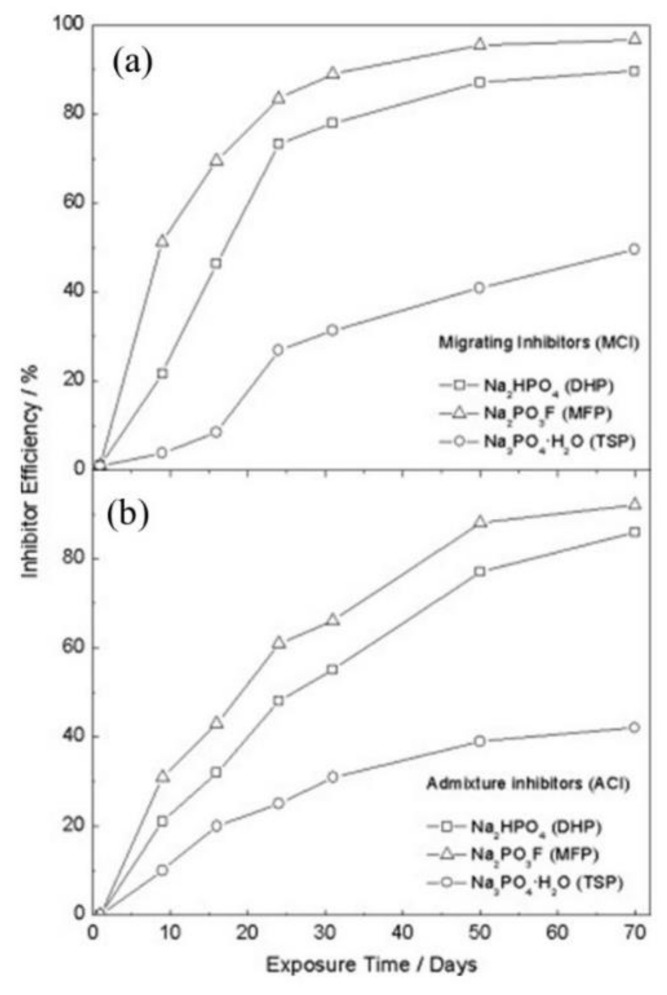
Inhibitor efficiency versus time for steel in the presence of: (**a**) migrating corrosion inhibitor (MCI), and (**b**) admixture corrosion inhibitor (ACI) [42]. Reproduced with permission from Bastidas, D.M. et al., Constr. Build. Mater.; published by Elsevier, 2010.

The corrosion inhibition efficiency (IE) over time for the embedded rebar in MCI and ACI is shown in Figure 20 [42]. The IE was obtained using Equation (3) [102]:

(3) $$\mathrm{IE}\;\left(\%\right)=\frac{\left(i_{\mathrm{corr},\mathrm{abs}}-i_{\mathrm{corr},\mathrm{pre}}\right)}{i_{\mathrm{corr},\mathrm{abs}}}\times 100$$

where *i*_corr,abs_ and *i*_corr,pre_ are the steel *i*_corr_ (estimated from LPR measurements) in the absence and presence of corrosion inhibitor, respectively. 

**Figure 21:**
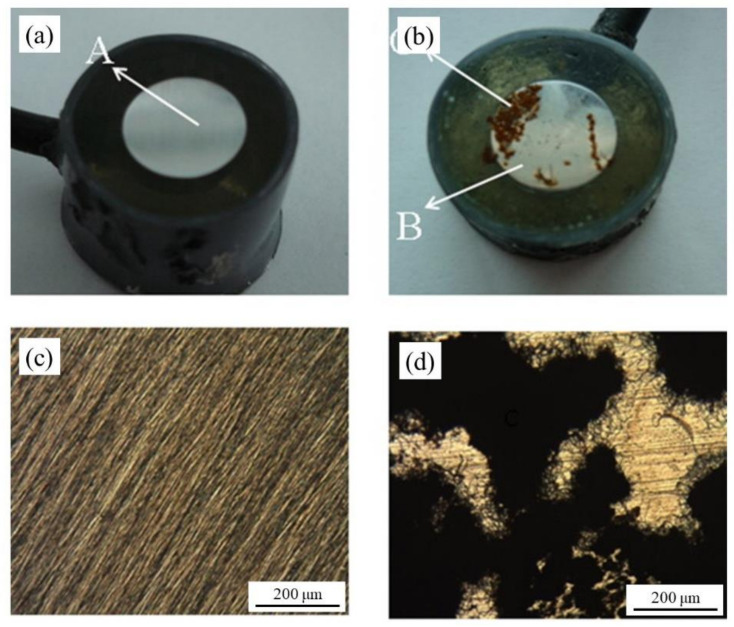
Micrographs of the electrodes after having carried out anodic polarization curves: (**a**) PSS + Cl^−^ + PO_4_^3−^, (**b**) PSS + Cl^−^ without and with corrosion products, (**c**) micrograph magnification of PSS + Cl^−^ + PO_4_^3−^, and (**d**) micrograph magnification of PSS + Cl^−^ without and with corrosion products [60]. Reproduced with permission from Yohai, L. et al., Electrochim. Acta; Published by Elsevier, 2013.

Overall, for the MCI specimens, the MFP compound showed the best IE. However, the MFP and DHP compounds showed the highest IE values after 50 days of exposure. The order of IE is MFP > DHP > TSP. The ACI specimens showed a similar trend to the MCI specimens (Figure 20a). As previously discussed with the enhancement of the corrosion properties seen by the EIS, by optical micrographs, it is unequivocal that the presence of PO_4_^3−^ helped maintain the integrity of the steel reinforcement, seen by the absence of corrosion (see Figure 21a,c) compared to the non PO_4_^3−^, which presented clear signs of dissolution (see Figure 21b,d) [60].

## 5. Thermodynamics and Reactivity of Phosphate Corrosion Inhibitors

The formation of iron phosphate compounds, Fe_3_(PO_4_)_2_ and FePO_4_ [103,104], see Equations (4) and (5), is thermodynamically favored over the formation of iron chloride compounds, FeCl_2_ and FeCl_3_ [105], see Equations (6) and (7), therefore leading to the formation of a stable phosphate barrier layer that can inhibit the chloride attack. Consequently, hindering the iron acid hydrolysis reaction [106], see Equations (8) and (9).

Fe^2+^ + 2 PO_4_^3−^ ⇆ Fe_3_(PO_4_)_2_   ∆*G*_f_°_Fe_3_(PO_4_)_2__ = −2444.80 kJ/mol (4)

Fe^3+^ + PO_4_^3−^ ⇆ FePO_4_    ∆*G*_f_°_FePO_4__ = −1663.98 kJ/mol (5)

Fe^2+^ + 2 Cl^−^ ⇆ FeCl_2_    ∆*G*_f_°_FeCl_2__ = −302.35 kJ/mol (6)

Fe^3+^ + 3 Cl^−^ ⇆ FeCl_3_    ∆*G*_f_^°^_FeCl_3__ = −668.11 kJ/mol (7)

Solutions of FeCl_2_ are moderately acidic, then the hydrated Fe^2+^ accepts only one hydroxyl ion from the aqueous electrolyte solution, see Equations (8) and (9) [107].

Fe^2+^ + 2 H_2_O ⇆ FeOH^+^ + H^+^       ∆*G*° = 46.02 kJ/mol (8)

Fe^2+^ + 2 H_2_O ⇆ Fe(OH)_2_ + 2 H^+^    ∆*G*° = 34.73 kJ/mol (9)

The penetration of phosphate MCI is compromised by the low solubility and precipitation of phosphate compounds, which react with the concrete matrix forming a solid phase that no longer provides a barrier layer of protection to the carbon steel rebar. The MCI specimens showed low phosphate penetration with 1114 µm, 380 µm and 126 µm for MFP, DHP and TSP, respectively, see Figure 12, due to the low porosity of the OPC paste and by the reaction of phosphate ions with portlandite (Ca(OH)_2_) in the OPC, forming porous apatite (Ca_5_(PO_4_)_3_(OH,F)). This apatite formation likely blocks the inhibitor penetration. 

The portlandite and MFP reaction was studied by combining a 0.3 M MFP aqueous solution with 2 mL of deionized water and 0.5 g calcium oxide (CaO) in a reactor [108]. This reaction’s yields were (Ca(OH)_2_), apatite (Ca_5_(PO_4_)_3_(OH,F)), fluorite (CaF_2_) and calcite (CaCO_3_) crystalline phases and amorphous phase, which were present in a large amount (~63%).

**Figure 22:**
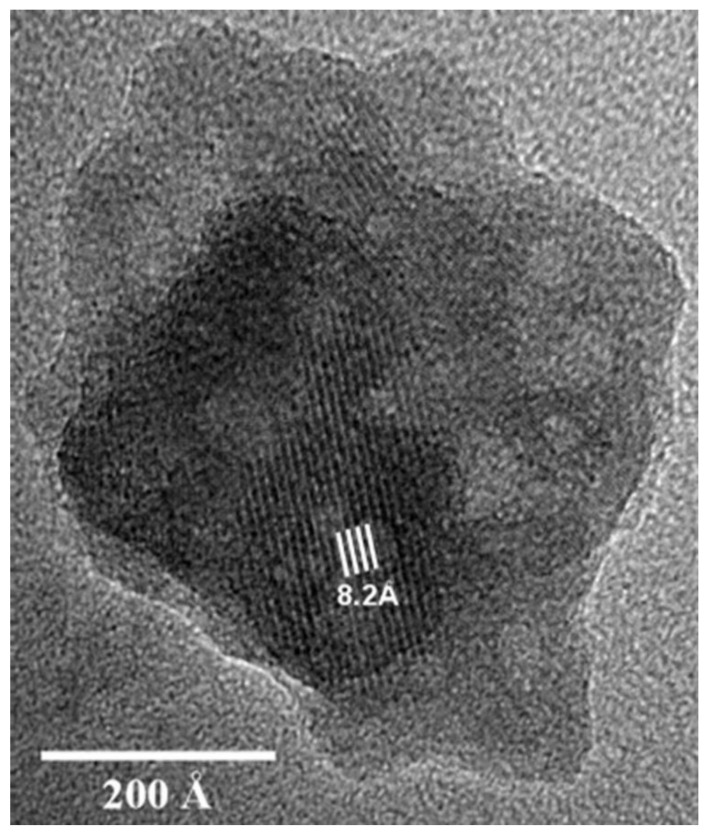
Lattice-fringe image showing *d*-spacing of 8.2 Å for an OPC mortar sample aged for 14 days [108]. Reproduced with permission from La Iglesia, A. et al., Constr. Build. Mater.; Published by Elsevier, 2012.

**Figure 23:**
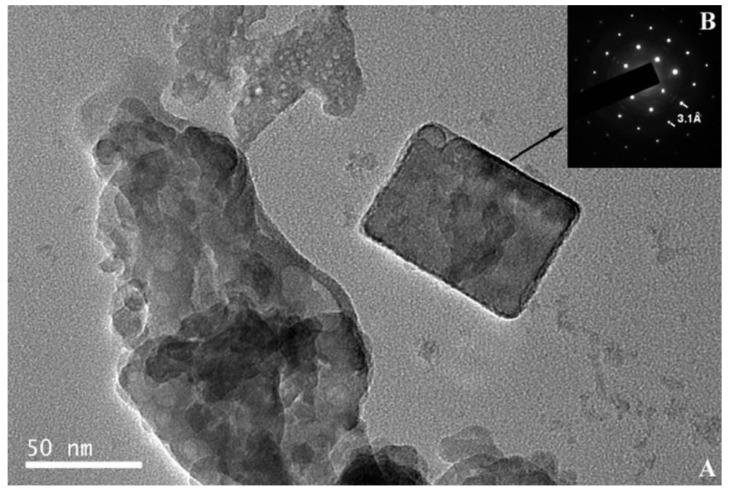
TEM image of a fluorite crystal for an OPC mortar sample aged for 6 h. Selected area electron diffraction pattern showing 3.1 Å *d*-spacing of (111) reflection [108]. Reproduced with permission from La Iglesia, A. et al., Constr. Build. Mater.; Published by Elsevier, 2012.

TEM images in Figure 22 and Figure 23 show the small crystals (50−60 nm) of apatite and ~50 nm crystal of fluorite with 8.2 Å and 3.1 Å spacing, respectively. This corresponds to (1 0 0) reflection of the apatite and (1 1 1) reflection of the fluorite. The half-life of apatite, fluorite and amorphous was 59.7, 40.8 and 48.1 days, respectively. The first step of the reaction is proposed to be a precipitation mechanism [108]. The amorphous phases are formed by the PO_4_^3−^, F^−^ and PO_3_F^2−^ ions and the dissolved Ca^2+^ ions from the Ca(OH)_2_, due to the high solubility product exceeding that of the crystalline calcium phosphate. 

Equation (10) shows the MFP dissolution process as it reacts with the pores of the portlandite (Ca(OH)_2_) substrate [61]:

6Ca(OH)_2_ + 3PO_3_F^2−^ + 6Na^+^ ⇆ Ca_5_(PO_4_)_3_F + CaF_2_ + 6OH^−^ + 6Na^+^ + 3H_2_O (10)

the pH of 12.4 of the portlandite suspension increased to 13.5 by the addition of MFP solution. The decrease in PO_3_F^2−^ ion activity and the consequent increase in the amount of PO_4_^3−^ ion generates the formation of hydroxyapatite (Ca_5_(PO_4_)_3_OH), Equation (11) [109,110]:

5Ca(OH)_2_ + 3PO_4_^3−^ + 9Na^+^ ⇆ Ca_5_(PO_4_)_3_OH + 9OH^−^ + 9Na^+^  ∆*G*_f_°= −152.71 kJ/mol (11)

The equilibrium constants for Equations (10) and (11) can be calculated by using the ∆*G*_f_° for the different species [109,110], thus the activities of the phosphate ions can be calculated for the MCI specimens, log(*a*_PO_3_F^2−^_) = −23.02 and log(*a*_PO_4_^3−^_) = −10.81. These low values confirm a low mobility for the phosphate ions in the OPC paste. Calcium monofluorophosphate (CaPO_3_F) or calcium hydroxide phosphate (CaPO_3_OH), however, can precipitate as amorphous phases and present a higher activity and mobility than the phosphate ions due to their increased solubility compared to the crystalline phases. The inconsistency between the diffusion for the compounds, see below (1.8 × 10^−8^ cm^2^/s for MFP, 6.7 × 10^−9^ cm^2^/s for DHP and 5.0 × 10^−9^ cm^2^/s for TSP) and the activities can be explained by the formation of calcium monofluorophosphate dihydrate from portlandite and monofluorophosphate (PO_3_F^2−^) ion [111,112], thus, according to Ostwald’s rule, fluorapatite is formed [113]:

Ca(OH)_2_ + PO_3_F^2−^ + 2Na^+^ + 2H_2_O ⇆ CaPO_3_F^−^ + 2H_2_O + 2OH^−^ + 2Na^+^ (12)

3Ca(OH)_2_ + 3CaPO_3_F⋅2H_2_O ⇆ Ca_5_(PO_4_)_3_F + CaF_2_ + 9H_2_O (13)

The equilibrium constant of Equation (12) and the activity of the monofluorophosphate (PO_3_F^2−^) ion can be calculated using the ∆*G*_f_° value for calcium monofluorophosphate dihydrate (CaPO_3_F·2H_2_O) of –2221.29 kJ/mol [114], log(*a*_PO_3_F^2−^_) = −4.66, explaining the high diffusion seen in OPC. Therefore, a precipitation−diffusion mechanism is proposed, in which the precipitation of the CaPO_3_F·2H_2_O phase occurs after the evolution of to Ca_5_(PO_4_)_3_F as depicted in Equation (13), greatly reducing the activity of the interstitial PO_3_F^2−^ ion.

Apatite formation may passivate the steel by the formation of a physical barrier of hydroxyl ions (OH^−^). These OH^−^ ions may diffuse through the Feldman−Sereda pore-network model [103], due to the lack of interaction of the PO_3_F^2−^ or PO_4_^3−^ ions with the silicate and portlandite. It is assumed that crystalline fluorapatite (Ca_5_(PO_4_)_3_F) is formed rather than amorphous phases. The volume variation (∆*V*) can be calculated in Equation (10) by using the different molar volumes (*V*_molar_): ∆*V* = *V*_molar(Ca_5_(PO_4_)_3_F)_ + *V*_molar(CaF_2_)_ − 6 *V*_molar(Ca(OH)_2_)_ = 24.69 Å^3^, which is a loss of 7.55% from the initial volume. Similarly, the volume loss from Equation (4) showed a decrease of 4.44%. These results suggest that the porosity of the cement is increased by some components, thus promoting the penetration of MFP. The contradiction of this description and the low penetration of MFP (1114 μm) can be explained by the deposition of fluorapatite and hydroxyapatite on portlandite, thus hindering further penetration.

The hybrid system formed by a tertiary amine with inorganic phosphorus compounds as a corrosion inhibitor for reinforcing steel yielded a capillary coefficient of ~3 g/m^2^ s^1/2^ for the inhibitor and ~6 g/m^2^ s^1/2^ for water, indicating that the inhibitor penetration was limited to the first 20 mm of concrete depth [29]. This behavior was associated with the high inhibitor viscosity compared to water and mainly a physicochemical interaction between the inhibitor mixture and the concrete pore surface leading to the formation of precipitated compounds that block the concrete porosity.

Overall, the addition of MFP, DHP, or TSP showed improvements on the corrosion inhibition of steel by the *E*_corr_ values. The change in *E*_corr_ shifted values in the anodic direction causing the system to be at a lower risk to corrosion for both MCI and ACI specimens, see Figure 15. However, the immersion electrochemical analysis showed a high risk of corrosion by *i*_corr_ values for specimens in distilled water and a low to medium risk level for MCI specimens, Figure 17, top. The ACI specimens in Figure 17b, showed that DHP presented the best inhibitive behavior, measuring *i*_corr_ values in the passive range for the duration of the test. The high initial *i*_corr_ values followed by a sharp decrease is suggestive of the protection provided by the inhibitor or alkaline environment or the pore network solution. The following increase in *i*_corr_ may be explained by the precipitation−diffusion mechanism.

DHP, MFP and TSP also show increased corrosion inhibition by *E*_corr_ shifting in the cathodic direction in the presence of 3.5 wt.% NaCl, see Figure 16. The MCI specimens (Figure 16a) showed corrosion risks of high, medium and low levels, while the ACI specimens (Figure 16b) showed medium or high risks. The MFP, DHP and TSP all can be categorized as cathodic inhibitors in 0.2 M (MCI specimens) or 3 wt.% (ACI specimens). However, other studies in literature have shown conflicting mechanisms with phosphates acting as cathodic inhibitors due to film precipitation on the substrate surface [41], or by a lower ratio of phosphate to chloride concentration [57]. Phosphates also serve as mixed corrosion inhibitors when present at high concentrations with seemingly no change in *E*_corr_ or an anodic inhibitor in the presence of chloride and oxygen [66], see Figure 7. MCI specimens, see Figure 19a, indicate low or medium corrosion risk and the superior corrosion inhibitor was determined to be MFP and DHP. For the ACI specimens, see Figure 19b, MFP presented the best performance with *i*_corr_ values near the passive state boundary. The MCI specimens showed *i*_corr_ values near the passive state for the majority of the test duration. Overall, the performance of the inhibitors can be placed in the following order from most effective to least effective; MFP, DHP and TSP, evaluated by IE. 

The phosphate diffusivity is calculated by the EPMA results (Figure 12) for all three MFP, DHP and TSP corrosion inhibitors in OPC. The PO_4_^3−^ is used to estimate the diffusion coefficient for each compound by measuring the content over the penetration depth based on the one-dimensional solution to Fick’s second law [115]. The effective diffusion coefficients (*D*) obtained were of 1.8 × 10^−8^ cm^2^/s for MFP, 6.7 × 10^−9^ cm^2^/s for DHP and 5.0 × 10^−9^ cm^2^/s for TSP, indicating a diffusion rate order of MFP > DHP > TSP. These values can be regarded as estimations due to the small penetration profile of 126 μm. The diffusion of phosphate decreases with time due to the formation of precipitates in the capillary network. Additionally, some ions will chemically bond as they form precipitates in the pore network. 

The ACI specimens showed average P_2_O_5_ contents of 0.47%, 0.45% and 0.43% for MFP, DHP and TSP, respectively, while the MCI specimens displayed ~0.10% for all three phosphate compounds. The small difference in P_2_O_5_ content was insufficient to significantly affect corrosion inhibition, as evidenced by the *i*_corr_ values within the same order of magnitude for the MCI and ACI specimens. Therefore, the ideal procedure for assessing the corrosion inhibition is not using a diffusion-based process with aqueous phosphate solutions. Mixing the phosphate compound with OPC cement paste can better prevent the corrosion process. To effectively apply this procedure, approximately 1% DHP can be added to protect the steel reinforcement and prevent the interaction of DHP and portlandite (Ca(OH)_2_). 

**Figure 24:**
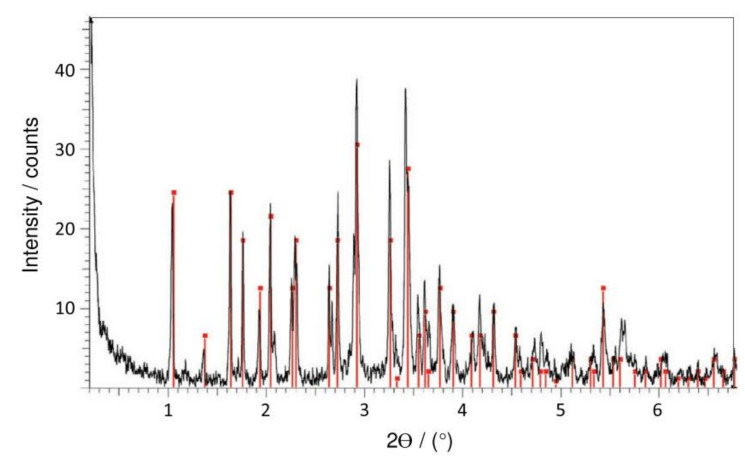
X-ray diffraction (XRD) pattern of the patina of sodium monofluorophosphate (Na_2_PO_3_F, MFP), showing canaphite (Na_2_CaP_2_O_7_·4H_2_O) phase represented by red peaks [43]. Reproduced with permission from Bastidas, D.M. et al., Cem. Conc. Comp.; published by Elsevier, 2013.

The mortar specimens displayed a white patina, 0.5−1.0 mm in thickness, formed after immersion in 5 wt.% Na_2_PO_3_F (MFP) solution (see Figure 7). The patina was mainly composed of canaphite (Na_2_CaP_2_O_7_·4H_2_O) as indicated by XRD analysis, see Figure 24, where the canaphite is represented by red (38–410 JCPDS file). It can be observed that the canaphite lines matched the experimental peaks. The pH of the phosphate solutions before immersion of the mortar specimens was 12.87 for Na_3_PO_4_·H_2_O, 7.75 for Na_2_HPO_4_ and 5.99 for Na_2_PO_3_F. After 40 days of immersion in the solution the pH was 12.78 for Na_3_PO_4_·H_2_O, 10.46 for Na_2_HPO_4_ and 11.26 for Na_2_PO_3_F, showing that the OPC mortar causes considerable alkalization of the MFP and DHP solutions but only small changes in the TSP solution. The pH of the medium is the parameter that determines the stability of an ionic species.

**Figure 25:**
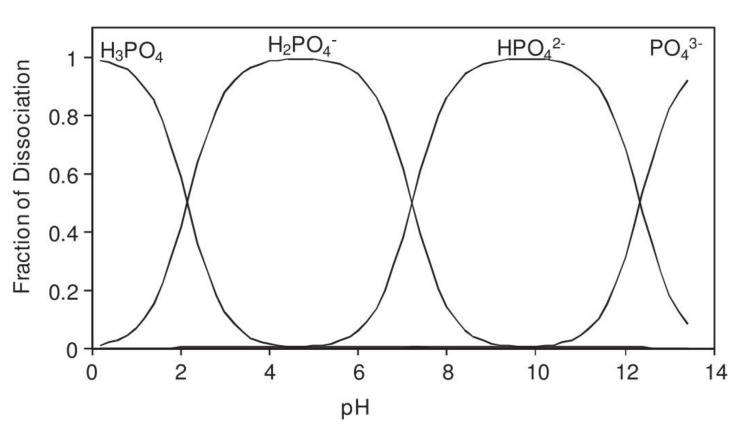
Distribution diagram for phosphoric acid (H_3_PO_4_) [43]. Reproduced with permission from Bastidas, D.M. et al., Cem. Conc. Comp.; published by Elsevier, 2013.

The mass balance of the phosphate species in solution may be determined using Equation (14):

[H_3_PO_4_] + [H_2_PO_4_^−^] + [HPO_4_^2−^] + [PO_4_^3−^] = C (14)

where C is the total phosphate ion concentration. The equilibrium constants of the acid solutions, *K*_a1_, *K*_a2_ and *K*_a3_ are defined as: p*K*_a1_ = 2.23, p*K*_a2_ = 7.21 and p*K*_a3_ = 12.32. The resulting four linear equations system can be solved to obtain the fraction of dissociated phosphate ionic species versus pH, see Figure 25. It can be observed that the H_2_PO_4_^−^ ion is stable in the pH range of 3–6, the HPO_4_^2−^ ion is stable in the pH range of 8–12, while the PO_4_^3−^ ion is stable above pH 12 [116].

In the same way, the degree of dissociation of monofluorophosphate was obtained considering the equilibriums of Equations (15) and (16):

H_2_PO_3_F ⇆ HPO_3_F^−^ + H^+^    *K*_a4_ = [H^+^][HPO_3_F^−^]/[H_2_PO_3_F] (15)

HPO_3_F^−^ ⇆ PO_3_F^2−^ + H^+^    *K*_a5_ = [H^+^][PO_3_F^2−^]/[HPO_3_F^−^] (16)

and the mass balance of the phosphate species in solution may be determined by Equation (17):

[H_2_PO_3_F] + [HPO_3_F^−^] + [PO_3_F^2−^] = C (17)

Monofluorophosphate acid solution equilibrium constants (*K*_a4_ and *K*_a5_) were calculated using the standard Gibbs free-energy of formation (∆*G*_f_°) values [110].

**Figure 26:**
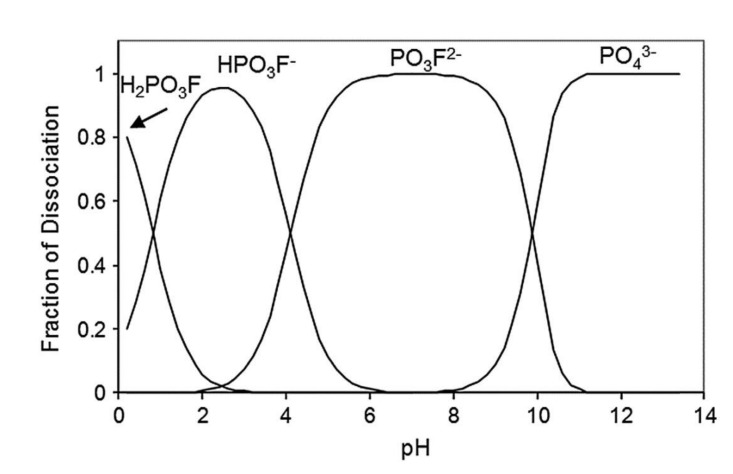
Distribution diagram for sodium monofluorophosphate (Na_2_PO_3_F) (MFP) [43]. Reproduced with permission from Bastidas, D.M. et al., Cem. Conc. Comp.; published by Elsevier, 2013.

Figure 26 shows the speciation diagram for the three species H_2_PO_3_F, HPO_3_F^−^ and PO_3_F^2−^ calculated as a function of the pH. In an alkaline medium the fluorophosphate (PO_3_F^2−^) ion may react with the hydroxyl (OH^−^) ion according to Equation (18):

PO_3_F^2−^ + 2 OH^−^ ⇆ PO_4_^3−^ + F^−^ + H_2_O    ∆*G*° = −54.34 kJ/mol (18)

where ∆*G*_f_° is the standard free-energy of reaction. From the ∆*G*° value, the equilibrium constant (*K*_eq_) of Equation (18) was calculated, yielding *K*_eq_ = 8.772 × 10^7^. Considering the equilibrium constant, the mass balance: [PO_4_^3−^] + [PO_3_F^2−^] = 1 and the parity in Equation (18): [PO_4_^3−^] = [F^−^], the following expression can be obtained:

[PO_4_^3−^]^2^ + 8.772 × 10^7^ [OH^−^]^2^ [PO_4_^3−^] − 8.772 × 10^7^ [OH^−^]^2^ = 0 (19)

Figure 26 includes the distribution of PO_3_F^2−^ and PO_4_^3−^ ions obtained by plotting the positive value solution of Equation (19) as a function of the pH. It can be concluded that the stable range for HPO_3_F^−^, PO_3_F^2−^ and PO_4_^3−^ ions is in a pH range of 2–4, 5−9 and above pH 11, respectively. Because the pH of the mortar should be above 12, the phosphate and fluorophosphate species are transformed to orthophosphate (PO_4_^3−^) ion.

**Figure 27:**
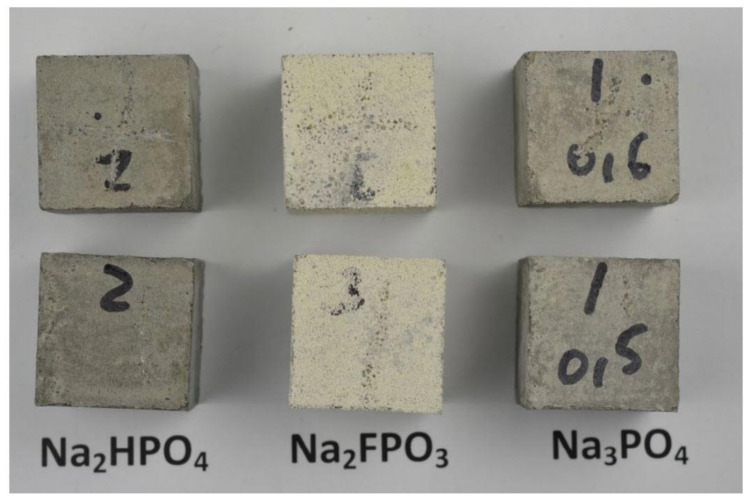
Specimens exposed to 5 wt.% Na_2_HPO_4_ (DHP), Na_2_PO_3_F (MFP) or Na_3_PO_4_ (TSP) for 40 days [43]. Reproduced with permission from Bastidas, D.M. et al., Cem. Conc. Comp.; published by Elsevier, 2013.

The PO_3_F^2−^ ion is shown to be chemically unstable in alkaline media, shown by the formation of the canaphite patina formed on the mortar surface in 5 wt.% MFP solution, see Figure 27. The development of the patina is described by the following process; (i) the dissolution of portlandite allows for the formation of an interface with a high concentration of Ca^2+^ and OH^−^, (ii) PO_4_^3−^ ions are formed at the interface from the destabilized PO_3_F^2−^ ions, (iii) new PO_4_^3−^ ions react with the residual PO_3_F^2−^ ions (PO_4_^3−^ + PO_3_F^2−^ ⇆ P_2_O_7_^4−^ + F^−^) with ∆*G*_f_° = −4.29 kJ/mol (spontaneous reaction) and (iv) the phase crystallizes when the Na^+^, Ca^2+^ and P_2_O_7_^4−^ ions concentration exceed the solubility product of canaphite. 

Phosphate compounds are known to precipitate at a wide range of pH levels [44]. According to Mandal et al. [117], in the presence of phosphates a passive layer is generated on steel containing oxides/hydroxides such as goethite (α-FeOOH) and akaganeite (β-FeOOH); both are thermodynamically very stable and sparingly soluble phases, lepidocrocite (γ-FeOOH) and maghemite (γ-Fe_2_O_3_) as well as the thermodynamic stable iron phosphate (FePO_4_). These oxides and oxyhydroxides are also generated on bare steel exposed to continental and marine environments [118]. Phosphate compounds inhibit steel corrosion by adsorption on goethite and other hydrated iron oxides, where the thermodynamic study showed that the adsorption of phosphate corrosion inhibitors is spontaneous and the phosphate molecules interact with the rebar surface by physisorption according to the Langmuir isotherm model [21,22,23].

## 6. Conclusions

The relevance of the findings described in the present review indicate that soluble phosphates react with portlandite to trigger the precipitation of an insoluble phosphate, thus reducing the phosphate content in the pore solution and, consequently, the capacity to act as a corrosion prevention method. Line profile EPMA analysis for phosphorous and fluorine showed similar behavior for the three soluble phosphates, penetrating the OPC mortar to a depth of more than 2 mm. Portlandite (Ca(OH)_2_), apatite (Ca_5_(PO_4_)_3_(OH,F)), fluorite (CaF_2_), calcite (CaCO_3_) and amorphous products were identified. The lower penetration of the inhibitors indicated by the authors (126–1114 µm) can be explained by the interaction between the inhibitor and the OPC paste. In the case of PO_3_F^2−^ ion, it has been found to be chemically unstable in alkaline media, shown by the formation of the canaphite patina formed on the mortar surface.

Overall, for the MCI specimens, the MFP compound showed the best IE. However, the MFP and DHP compounds showed the highest IE values after 50 days of exposure. The order of IE is MFP > DHP > TSP. The ACI specimens showed a similar trend to the MCI specimens. The formation of iron phosphate compounds, Fe_3_(PO_4_)_2_ and FePO_4_ with ∆*G*_f_° values of −2444.80 kJ/mol and −1663.98 kJ/mol, respectively, is thermodynamically favored rather than the formation of iron chloride compounds, FeCl_2_ and FeCl_3_ showing ∆*G*_f_° values of −302.35 kJ/mol and −668.11 kJ/mol, respectively. Therefore, leading to the formation of a stable phosphate barrier layer can inhibit the chloride attack, consequently, hindering the iron acid hydrolysis reaction.

## Figures and Tables

**Table 1:** Evaluation of corrosion rate of carbon steel in concrete [65]. Reproduced with permission from Millard, S. et al., NDT E Int.; Published by Elsevier, 2001.

Corrosion Rate	*R*_p_, kΩ cm^2^	*i*_corr_, µA/cm^2^	Mass Loss, µm/Year
Very High	2.5−0.25	10−100	100−1000
High	25−2.5	1–10	10–100
Low	250−25	0.1–1	1–10
Passive	>250	<0.1	<1

**Table 2:** Corrosion potential (*E*_corr_, mV vs. SCE) and corrosion current density (*i*_corr_, µA/cm^2^) for different soluble phosphates: trisodium phosphate (Na_3_PO_4_) (TSP), tetrasodium pyrophosphate (polyphosphate) (Na_4_P_2_O_7_) (TSPP), hydroxyethylidene-diphosphonic (C_2_H_8_O_7_P_2_) (HEDP) and sodium monofluorophosphate (Na_2_PO_3_F) (MFP), exposed to simulated concrete pore solution (SCPS) and concrete matrix contaminated with Cl^−^.

Phosphate Inhibitor	Electrolyte	*E*_corr_ (mV_SCE_)	*i*_corr_ (μA/cm^2^)	Reference
TSP	SCP solution	−519	<0.1	[8]
	0.1M NaCl 0.1M NaCl + TSP	−450 −350	0.1 0.07	[38]
	Blank solution (SCP) SCP + 0.5M NaCl + 0.5M TSP	−500 −250	5600 700	[45]
	Portland mortar Portland mortar + Cl^−^ Portland mortar + Cl^−^ + TSP	−550 −400 −500	1300 1300 520	[56]
	Portland concrete	−100	<0.1	[57]
	Portland mortar	−650	5200	[58]
	Blank solution (SCP) SCP + Cl^−^, [Cl^−^]/[OH^−^] = 3 SCP + Cl^−^ + PO_4_^3−^, [PO_4_^3−^]/[Cl^−^] = 1	−208 −237 −263	0.006 14.0 0.02	[60]
	Blank solution (SCP) SCP + TSP	−200 −500	10.0 0.1	[62]
	Portland mortar + TSP	−246	0.1	[63]
	Blank solution (SCP) SCP + Cl^−^, 3% NaCl SCP + Cl^−^ + TSP	−180 −540 −430	0.1 10 1.0	[66]
TSPP	SCP + Cl^−^ + TSPP	−500	9	[66]
HEDP	SCP + Cl^−^ + HEDP	−520	9	[66]
MFP	Portland concrete + 0.6% Cl^−^ Carbonated Portland concrete + 0.6% Cl^−^	− −	<0.1 >0.1	[15]
	Portland mortar + (0.5−0.8%) Cl^−^	−450	>0.1	[61]

**Table 3:** Chemical composition (wt.%) of tested ordinary Portland cement (OPC).

CaO	SiO_2_	Al_2_O_3_	Fe_2_O_3_	MgO	MnO	TiO_2_	K_2_O	Na_2_O	SO_3_	LOI *	IR **
57.84	20.33	3.40	4.68	1.51	0.10	0.09	0.72	0.51	7.26	3.42	1.23

* LOI: loss of ignition; ** IR: insoluble residue.

**Table 4:** Penetration depth for P and F in mortar specimens immersed in 5 wt.% Na_3_PO_4_·H_2_O (TSP), Na_2_HPO_4_ (DHP) and Na_2_PO_3_F (MFP) solution, water/cement (w/c) ratios of 0.5 and 0.6.

Penetration Depth (mm)						
Element	TSP w/c 0.5	TSP w/c 0.6	DHP w/c 0.5	DHP w/c 0.6	MFP w/c 0.5	MFP w/c 0.6
P	0.88	1.10	0.36	0.64	1.33	1.23
F	–	–	–	–	1.36	1.40

## Data Availability

Not applicable.

## Abbreviations

ACI	Admixed corrosion inhibitor
*D*	Diffusion coefficient
EDX	Energy dispersive X-ray
*E*_corr_	Potential of corrosion
*E*_PZC_	Potential of zero charge
EPMA	Electron probe micro-analysis
∆*G*_f_°	Standard free-energy of formation
*i*_corr_	Corrosion current density
IE	Inhibitor efficiency
*K*_a_	Acid dissociation constant
*K*_eq_	Equilibrium constant
LPR	Linear polarization resistance
MCI	Migrating corrosion inhibitor
OPC	Ordinary Portland cement
RH	Relative humidity
RT	Room temperature
*R*_p_	Polarization resistance
SCE	Saturated calomel electrode
SCPS	Simulated concrete pore solution
TEM	Transmission electron microscopy
TGA	Thermogravimetric analysis
*V*_molar_	Molar volume
∆*V*	Variation in volume
XRD	X-ray diffraction
